# Inhibitory Potential of the Truncated Isoforms on Glutamate Transporter Oligomerization Identified by Computational Analysis of Gene-Centric Isoform Maps

**DOI:** 10.1007/s11095-024-03786-z

**Published:** 2024-11-01

**Authors:** Alper Karagöl, Taner Karagöl, Mengke Li, Shuguang Zhang

**Affiliations:** 1 Istanbul University Istanbul Medical Faculty, Istanbul, Turkey; 2 State Key Laboratory of Microbial Metabolism, Joint International Research Laboratory of Metabolic and Developmental Sciences, and School of Life Sciences and Biotechnology, Shanghai Jiao Tong University, Shanghai, 200240 China; 3 Laboratory of Molecular Architecture, Media Lab, Massachusetts Institute of Technology, 77 Massachusetts Avenue, Cambridge, MA 02139 USA

**Keywords:** alternative splicing, integral membrane proteins, membrane protein design, protein 3D structural predictions, truncated isoforms

## Abstract

**Objective:**

Glutamate transporters play a key role in central nervous system physiology by maintaining excitatory neurotransmitter homeostasis. Biological assemblies of the transporters, consisting of cyclic homotrimers, emerge as a crucial aspect of glutamate transporter modulation. Hence targeting heteromerization promises an effective approach for modulator design. On the other hand, the dynamic nature of transcription allows for the generation of transporter isoforms in structurally distinct manners.

**Methods:**

The potential isoforms were identified through the analysis of computationally generated gene-centric isoform maps. The conserved features of isoform sequences were revealed by computational chemistry methods and subsequent structural analysis of AlphaFold2 predictions. Truncated isoforms were further subjected to a wide range of docking analyses, 50ns molecular dynamics simulations, and evolutionary coupling analyses.

**Results:**

Energetic landscapes of isoform-canonical transporter complexes suggested an inhibitory potential of truncated isoforms on glutamate transporter bio-assembly. Moreover, isoforms that mimic the trimerization domain (in particular, TM2 helices) exhibited stronger interactions with canonical transporters, underscoring the role of transmembrane helices in isoform interactions. Additionally, self-assembly dynamics observed in truncated isoforms mimicking canonical TM5 helices indicate a potential protective role against unwanted interactions with canonical transporters.

**Conclusion:**

Our computational studies on glutamate transporters offer insights into the roles of alternative splicing on protein interactions and identifies potential drug targets for physiological or pathological processes.

**Supplementary Information:**

The online version contains supplementary material available at 10.1007/s11095-024-03786-z.

## Introduction

Glutamate transporters are a class of membrane proteins that play a critical role in the central nervous system by removing excess glutamate from the synapse, which is the predominant excitatory neurotransmitter in the vertebrate central nervous system [1–3]. The synaptic effects of glutamate are terminated by the action of excitatory amino acid transporters (EAATs) located on the plasma membrane of astrocytes and neurons [1, 2]. This reuptake helps to prevent the excitotoxicity and the subsequent cell death that can occur as a result, which makes glutamate transporters crucial for neuronal physiology [2–4]. Consequently, dysregulations of glutamate transporters have been linked to the etiologies of many conditions, including neurodegenerative diseases [4], affective disorders [5], cancer [6], and Parkinson disease (PD) [7]. Biological assemblies of the glutamate transporters are cyclic homotrimers [1, 7, 8]. This formation emerges as a crucial aspect of glutamate transporter modulation. Depending on the transporter, trimerization can influence membrane trafficking, function, regulation, and turnover [2, 9–13]. Furthermore, studies have revealed that inhibition of homomeric configurations has an effect on transporter efficiency [10–12]. For instance, co-expression of EAA1 and its variant EAA1 ex9skip significantly reduces glutamate uptake activity, likely due to their interaction in the endoplasmic reticulum [10]. In cases of transporter dysregulation resulting in fatal disorders [1–7], modulation of trimerization may offer an effective approach to intervention.

The dynamic nature of transcription, where specific exons may be included or excluded, allows for the generation of structurally and functionally distinct proteins [14]. Alternative splicing, initiation, and promoter usage introduce a re-distribution in the coding regions of mRNA transcripts (exons), leading to the generation of multiple isoforms for a single transporter gene. The truncated isoforms lack specific exons for transport process but may persist in the structural similarity to the canonical transporter, especially the TM (transmembrane) helices contributing to trimerization. Hence, we propose they may interact with canonical transporters, similar to EAA1 ex9skip [10]. This mechanism was previously proposed for GPCRs, as we previously discovered that truncated receptors could function as negative regulators when co-expressed with the full-length counterpart [15–17]. In the same way, the isoforms may also form homodimers. This result in self-assembly of truncated isoforms that might counter their inhibitory effects on canonical transporters by competitive modulation.

In the context of membrane proteins, accurately predicting the contact surface is a challenging process. Subsequently, we benchmarked the data generated from conventional protein–protein docking methods (ClusPro, ClusPro multimer, RosettaDock, LightDock) and neural network based AlphaFold-Multimer, utilizing existing experimental structures of EAA1-3. We employed a multi-step approach to identify potential isoforms, considering their instability and structural characteristics, starting from gene-centric isoform maps. The large number of stable truncated isoforms identified was further subjected to comparative structural analysis and complex predictions. The predicted isoform-canonical transporter complexes were then re-ranked, and complex model generation was followed by building membrane systems for the complexes. Molecular dynamics simulations, trajectory analyses, and binding free energy calculations quantified the tendency of truncated isoforms to form complexes with canonical transporters. Decomposition analysis of contributing residues was conducted to explore the potential for residue-specific targeting in therapeutic interventions.

In this study, we present a comprehensive analysis of isoforms and alternative pre-mRNA splicing of the glutamate transporter subfamily. Our findings reveal an inhibitory potential of trimerization of EAA1 and EAA2 by truncated isoforms, with isoforms mimicking TM1-5 canonical helices predicted to interact with canonical transporters. We also produce insights into the impact of self-assembly on the heteromerization process, offering a framework for computational studies. The proposed glutamate transporter heteromerization by truncated isoforms introduces a new layer to our understanding of synaptic regulation and identifies potential therapeutic interventions.

## Results and Discussions

### Canonical Binding Interface

**Fig. 1:**
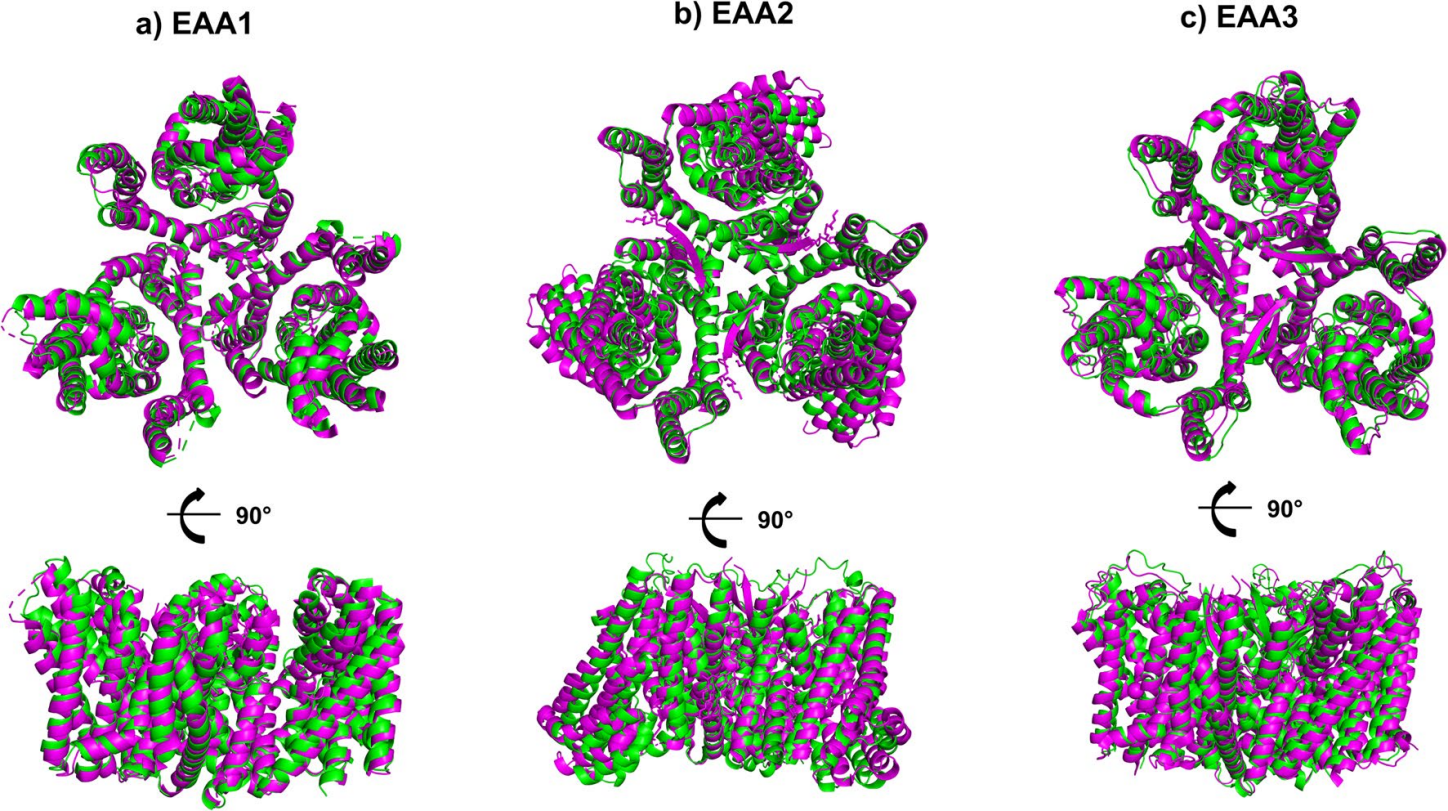
Superimposed experimental structures of EAA1, EAA2 and EAA3 biological assemblies with homotrimers predicted by AlphaFold2 Multimer. Structural data for native transporters (PDB: 5LLU 3.32Å), (7VR8 3.58Å), (8CV2 2.44Å) was acquired from the Protein Data Bank. The biological assemblies comprise homotrimer units. The experimental structures (magenta) EAA1^crystal^ (**a**), EAA2^EM^ (**b**), EAA3^EM^ (**c**) are superposed with AlphaFold2 multimer predicted homotrimer structures (green). The closest matches are visualized, with root mean square deviation (RMSD) values of EAA1 (3.08Å), EAA2 (2.82Å) and EAA3 (1.67Å). Large loops are removed for clarity to facilitate direct comparisons.

**Fig. 2:**
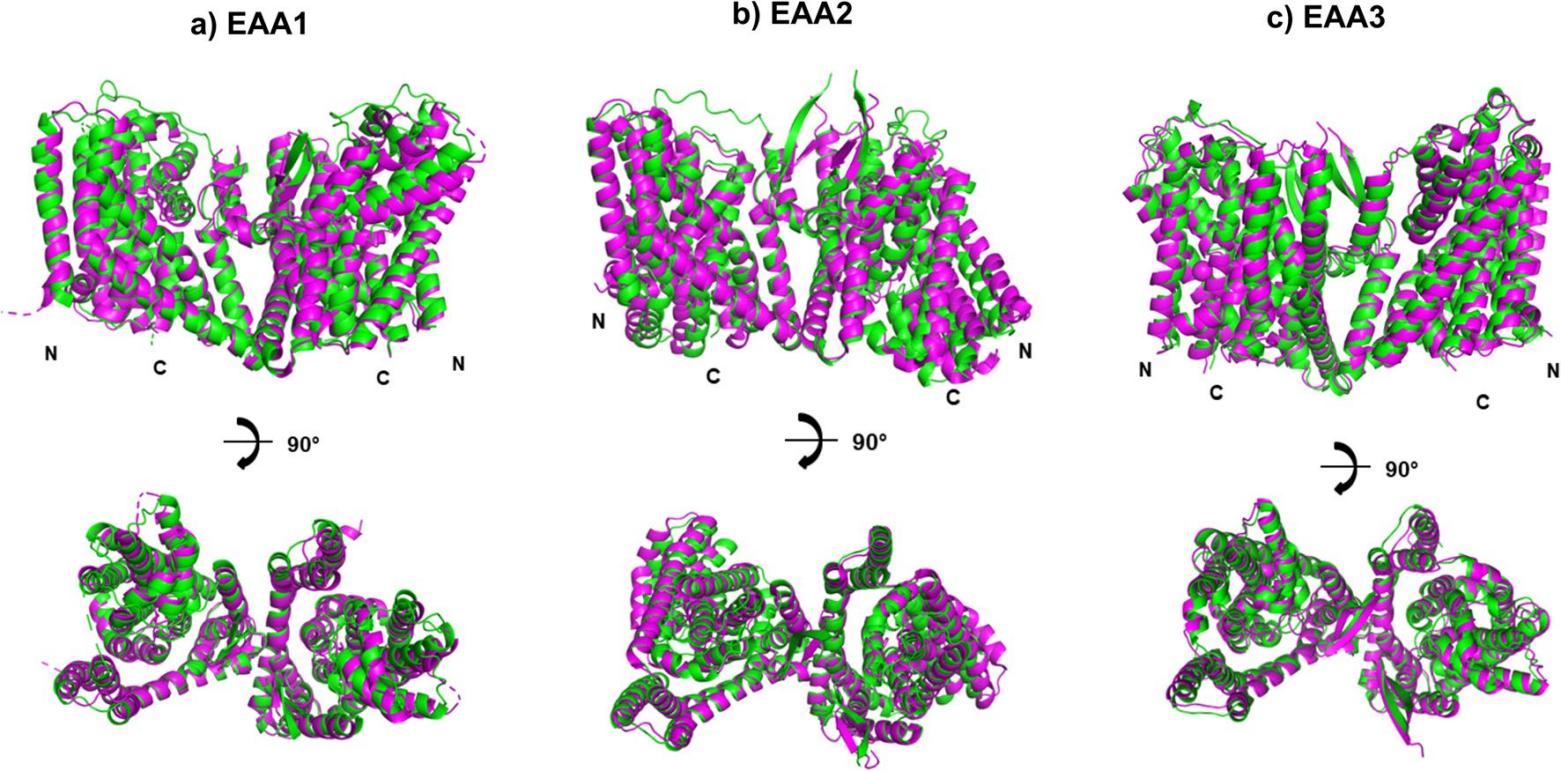
Superposed experimental structures of EAA1, EAA2 and EAA3 with AlphaFold2 multimer predicted homodimers. Structural data for native transporters (PDB: 5LLU 3.32Å), 7XR6 3.40Å, 8CV2 2.44Å was acquired from the Protein Data Bank. The biological assembly contains homotrimer, N- and C-termini are labelled. The experimental structures (magenta) EAA1^crystal^ (**a**), EAA2^EM^ (**b**), EAA3^EM^ (**c**) are superposed with AlphaFold2 multimer predicted homodimer structures (green). The closest matches are visualized, the RMSD values are EAA1 (2.97Å), EAA2 (2.67Å) and EAA3 (1.42Å). Large loops and one unit of the homotrimer are removed for clarity to facilitate direct comparisons.

The biological assemblies of EAATs correspond to cyclic homotrimers [6, 9, 13] (Fig. 1). The canonical monomer consists of eight α-helical transmembrane regions (TM1-TM8). While TM2, TM4, and TM5 mediate inter-monomer contacts in the trimer, the entire TM1-TM6 segment is referred to as the "trimerization domain” [13]. Our AlphaFold multimer predictions aligned with experimental homotrimer structures of EAA1-3, revealing an interface composition of TM2, TM4, and TM5, with additional contributions from N- and C-termini helices and parts of Extracellular Loop 1 (ECL1). Compared with the experimental assemblies, the RMSD values of AlphaFold predicted dimers were 2.97Å, 2.67Å, 1.42Å, respectively for EAA1-3 (Fig. 2). The contact surface of experimental structures indicates a strong interaction between TM4-TM5 alpha-helices and TM2-TM3 domains. Interestingly, these motifs were found to be phylogenetically conserved in our previous study involving glutamate, vesicular glutamate, and monoamine transporters [18, 19]. The conservation of trimerization domains (TM2, TM4, TM5) and interaction surfaces highlights the importance of these regions in maintaining functional trimer formation and substrate transport.

### Helical Composition of the Truncated Isoforms

To explore potential modulatory effects, the isoforms were speculated to have a structural resemblance to the corresponding canonical trimerization interface (Supplementary Figure S1). Structural superposition quantified the similarity of isoforms (Supplementary Table S1), suggesting a conservation of helical motifs among the sampled isoforms (Table I). The helices in canonical transporters were previously calculated to be evolutionarily conserved, greatly surpassing the conservation scores of loops and N- and C-termini [18].

**Table I:** Sampled Protein Coding Isoforms of EAA1, EAA2, and EAA3

Name	Isoform ID^1^	Length^2^	TM count^3^	TM composition^4^
EAA1 (SLC1A3)	P43003 (Canonical EAA1)	542	8	Canonical
	E7EUV6	106	2	TM1, TM2
	A0A7P0T9Z4	123	3	TM6, HP1, HP2
EAA2 (SLC1A1)	P43004 (Canonical EAA2)	574	8	Canonical
	C9J9N5	409	7	TM1, TM2, TM3, TM4, TM5, TM6, HP1
	A0A2R8Y642	435	4	TM2, TM3, TM4, TM5
	A0A2R8Y4N0	330	5	TM5, TM6, HP1, TM7, HP2
EAA3 (SLC1A2)	P43005 (Canonical EAA3)	524	8	Canonical
	H0Y7R2	242	2	TM5, TM6

^1^The Uniprot entry ID of the isoform (only included isoforms that have length between 15 and 85% of the canonical sequence). Isoforms that are excluded from the sample are not included in this table (Please see Supplementary Table 1)

^2^The length of the aminoacid sequence

^3^Transmembrane (TM) domain count of the protein, derived from the topology information included in the Uniprot entries

^4^Transmembrane (TM) coposition of the protein, derived from the topology information included in the Uniprot entries. TM helices are listed according to canonical transporter

E7EUV6 retains only the first two TM domains (N-termini) of the canonical isoform of EAA1. Whereas A0A7P0T9Z4 Isoform includes TM6 and two hairpin helices (HP1, HP2), which mimic the latter part (C-termini) of the canonical EAA1. Thus, it may engage in different interactions compared to the E7EUV6. This may also exert influence on binding to canonical monomer: A0A7P0T9Z4 of EAA1 had lesser extent of trimerization contributing helices of canonical protein and E7EUV6 contains only a part of the trimerization domain (specifically the TM2 domain). In the case of the truncated isoforms encoded by the EAA2 gene (= SLC1A2), the near-complete TM structure suggests a high level of functional mimicry, likely retaining most binding characteristics of the canonical transporter with minor deviations. The A0A2R8Y642 isoform had four TM domains mimicking the central TM regions (TM2, TM3, TM4, TM5) of canonical EAA2. The A0A2R8Y4N0 isoform conserves the C-termini, further incorporating TM5 helices. The H0Y7R2 isoform of EAA3 retains the TM5 and TM6 domains of the canonical transporter.

The variations in Root Mean Square Deviation (RMSD) values among the isoforms of EAA1, EAA2, and EAA3 quantify the structural deviation from their respective canonical transporters. The EAA1 and EAA3 truncated isoforms exhibit deviations of 3Å, with the total deletion of remaining helical residues (Supplementary Figure S2). Conversely, EAA2 isoforms are more structurally similar to the canonical form (Supplementary Table S1), with the truncated isoforms of EAA2 retaining a larger part of the canonical trimerization domains (Table I). This indicates that neighboring helices play a supporting role in the structural similarity to the truncated isoforms. The observed structural conservation of critical regions and similarities suggests an interaction with canonical transporters, as they contain helical domains contributing to trimerization. This may also have evolutionary significance, as our previous study of dopamine, serotonin, and norepinephrine transporters showed that the structural similarity of helices might act as a selection pressure independent of solubility [19].

### Expression Patterns and Other Characteristics

Glial EAA2 is primarily responsible for removing excess glutamate, whereas the other four EAATs have more locally restricted activities [1, 20, 21]. It was also identified that many disease-associated variants affect the efficiency of translation of EAA2 in AD and ALS [21–23]. Unaltered EAA2 mRNA levels indicate that EAA2 expression levels are controlled at post-transcriptional level, while the mechanism underlying the loss of EAA2 is unclear [21]. As shown in studies involving the EAA1ex9skip variant, truncated isoforms were able to reduce the insertion of the canonical transporter into the membrane [10]. Correlated with their distinct expression patterns, we identified various EAA2 isoforms mimicking canonical helices from the analysis of gene centric mapping (Table I).

In contrast to EAA1 and EAA2, EAA3 is mostly intracellular and has a rapid regulation of its membrane trafficking [1, 24, 25]. Due to its ability to transport cysteine, EAA3 is also crucial for protection from oxidative stress [24]. At the same time, only modest changes in the expression of EAA3 occur with age, contrary to EAA1-2 [1, 26, 27]. EAA3 was shown to be primarily regulated by intracellular trafficking rather than by changes in mRNA expression levels [1]. Interestingly, EAA3 expression characteristics are also correlated with EAA3 has fewer distinct isoform transcripts compared to EAA1 and EAA2 (Tables I and II). Further empirical evidence is beneficial to substantiate the expression patterns of isoforms and their potential impact on transporter dynamics. Additionally, we further hypothesized that the self-assembly of truncated isoforms may also counter their inhibitory effects on canonical transporters by competitive modulation (by having more affinity to binding themselves rather than canonical transporters). The sampled complexes (isoform-isoform and isoform-canonical) in this study exhibited diversity in terms of topology, TM regions, and sequence lengths.

**Table II:** Expression Patterns of Glutamate Transporter Subtypes

Gene Name	Canonical Product	Function^1^	Primary Expression^2^	Regional specificity^3^
SLC1A3	EAA1	Glutamate transporter in astrocytes	Brain (Astrocytes)	Cerebellum
SLC1A2	EAA2	Glutamate transporter in astrocytes and axon terminals	Brain (Astrocytes) axon terminals (presynaptic)	None
SLC1A1	EAA3	Neuronal glutamate and cysteine transporter	Brain (Neurons) Kidney Intestine Liver	None

^1,2,3^This table is mainly derived from Malik *et al*. [1]

### Benchmarking with Experimental Data

Benchmarking with experimental data consisted of experimental canonical models of EAA1, EAA2 and EAA3, and predicted models with five computational methods, namely, AlphaFold multimer, ClusPro, ClusPro multimer, LightDock and RosettaDock (105 models for the benchmarking). AlphaFold multimer predictions were more accurate than the conventional docking methods utilized in our sample of glutamate transporters, with RMSD as close as 2Å compared to experimental docking data derived from X-ray and cryo-EM (Supplementary Table S2). This is followed by RosettaDock5 predictions with RMSDs of median (M) = 10.6Å. Meanwhile, the complexes generated by ClusPro (M = 20.7Å), ClusProMultimer (M = 15Å), and LightDock (M = 28Å) had higher RMSD deviations from the experimental structures. Since AlphaFold multimer models for canonical complexes were observed to be substantially more accurate than remaining conventional docking algorithms, they are preferred for isoform complex predictions through this study, with additional model re-ranking. The AlphaFold algorithm is prone to produce biases towards canonical interfaces, when analyzing isoform structures [28]. This is also previously mentioned by our studies analyzing coevolution data of AlphaFold output [19]. To reduce the excess dependence on templates, no template mode was utilized, and structures were re-ranked.

### Model Re-ranking

In this study, the single-point energy predictions were conducted for screening the models with optimal affinity. MMGBSA (Molecular Mechanics with Generalized Born Surface Area) calculations were preferred since the correct ranking of initial models was emphasized. The calculations included each AlphaFold2 output of the best 5 models per complex, thereby ensuring structural accuracy. The screening analysis indicates that E7EUV6, C9J9N5 and A0A2R8Y642 were good candidates for inhibitions of canonical transporter assemblies, as their heterodimers were ranked similar to canonical dimers (Supplementary Tables S3, S4).

Furthermore, the screening analysis indicates that lower ranked A0A2R8Y4N0 and H0Y7R2 isoforms have strong self-assembly tendencies, as they ranked higher than their complexes with canonical transporters (Supplementary Tables S5, S6). Meanwhile, A0A7P0T9Z4 of EAA1 had no self-assembly tendency nor binding to canonical EAA1. This suggests that TM5-TM6 helices were more indicative of self-binding than N-termini helices of TM1 and TM2. The trimerization process may create a steric hindrance, thus it could make it difficult for truncated isoforms to access binding sites on the canonical transporters. However, no distinct differences were observed for the majority of the isoforms in the screening analysis, since the contact surface of the dimers was also contributing to trimerization domain (Supplementary Tables S7, S8). Contrary to other isoforms, E7EUV6 containing heterotrimer had 26% decrease in single-point affinity compared to heterodimer complex. Further analysis would be beneficial to assess the exact effect of trimerization.

### Molecular Dynamic Simulations of the Membrane Systems

The trajectory profiles of isoform complexes during 50ns MD simulation were analyzed to discern the magnitude of fluctuations at each residue position within the glutamate transporters. The root-mean-square-fluctuation (RMSF) was around 2.5Å for the transmembrane helices (Supplementary Figure S3), indicating good stability of the complexes. We identified some flexible regions that are located in loops of both isoforms and canonical transporters. Remarkably, all isoform complexes exhibited a similar pattern of residue-wise RMSF as canonical dimers, while E6EUS7 isoform reduced flexibility after 10ns molecular dynamics (Supplementary Figure S4). Additionally, the radius of gyration was maintained through the molecular dynamics, and the calculated scores demonstrated a similar amplitude of vibrational dynamics compared to canonical dimers (Supplementary Figure S5). Notably, the isoform-EAA2 complexes have slightly increased solvent-accessible surface area (SASA) compared to canonical transporters (~ 480 to 460) (Supplementary Figure S6). This may have indications since alterations in SASA may impact the binding pocket accessibility and the substrate specificity of individual transporters, thus inhibiting the transport process [29].

**Fig. 3:**
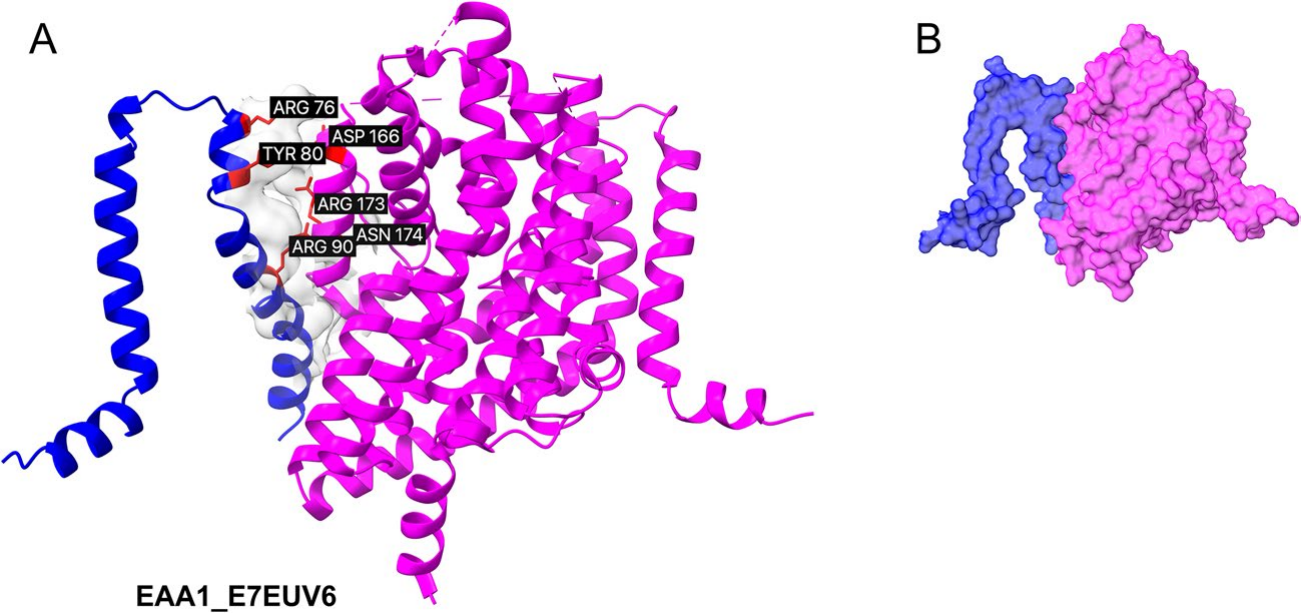
Heterodimeric complex containing canonical structure (EAA1) and the truncated isoform E7EUV6. Superposed canonical experimental transporter (magenta) and isoform structures (blue) are visualized. (**A**) The interface surface is displayed, with the six (three for the ligand and three for the receptor) highest energy-contributing residues highlighted in red and labeled. (**B**) The proposed competitive modulation of dimerization by the truncated isoform E7EUV6. The protein–protein complexes are predicted using AlphaFold2 multimer. The binding free energy for the isoform canonical complex is predicted as -15.66kcal/mol. Large loops are removed to facilitate direct comparisons.

**Fig. 4:**
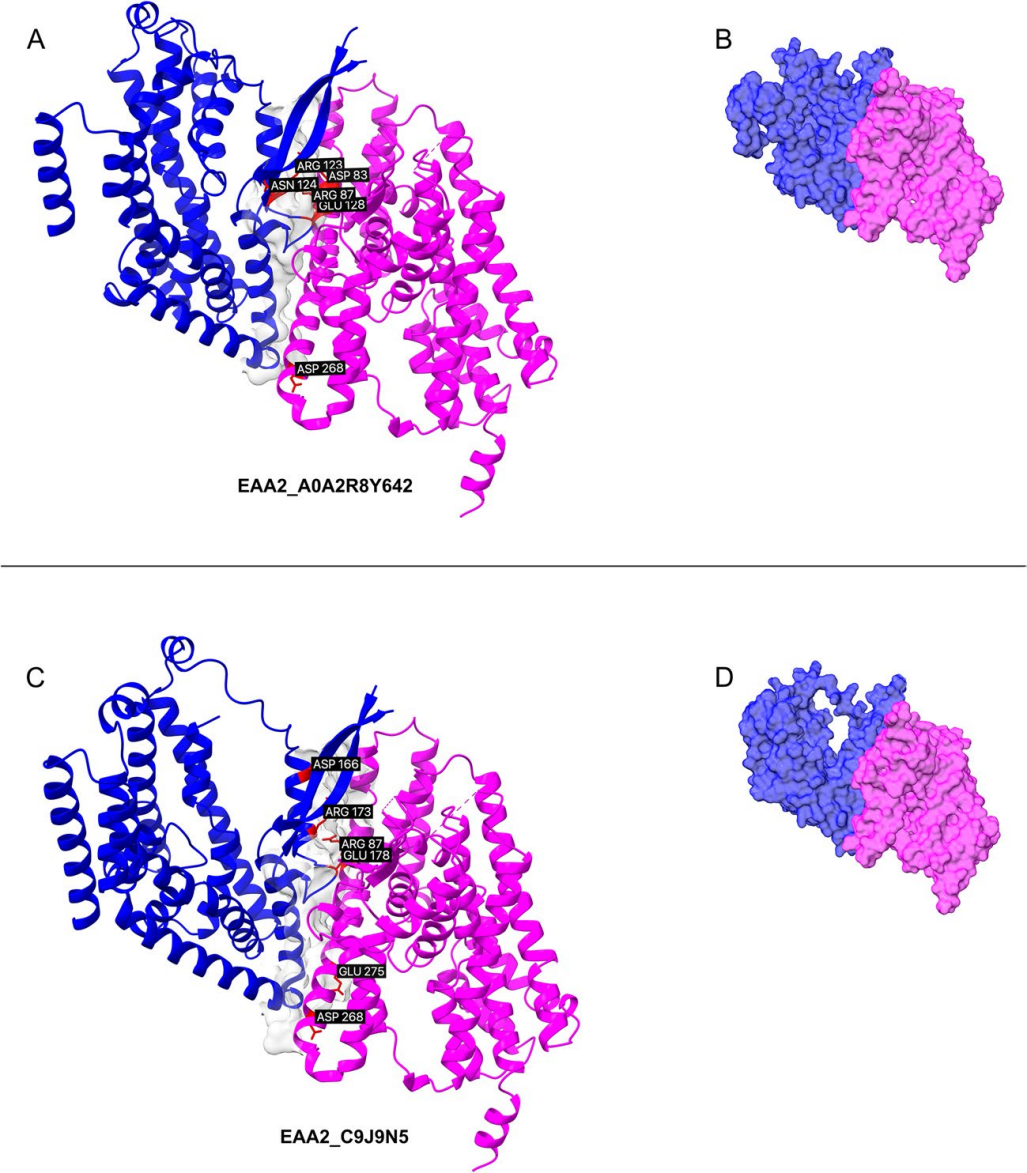
Complexes containing canonical structure (EAA2) and truncated isoforms. Superposed canonical experimental transporter (magenta) and isoform structures (blue) are visualized. (**A**, **C**) The interface surface is displayed, with the six (three for the ligand and three for the receptor) highest energy-contributing residues highlighted in red and labeled. (**B**) The competitive modulation of dimerization by the truncated isoform A0A2R8Y642, with binding free energy calculated as -31.00 kcal/mol. (**D**) The comparative modulation of dimerization by the truncated isoform C9J9N5. The protein–protein complexes are predicted with AlphaFold2 multimer. The binding free energy is -32.05 kcal/mol. Large loops are removed for clarity to facilitate direct comparisons.

In this study, Molecular Mechanics Poisson-Boltzmann Surface Area (MMPBSA) calculations were conducted on membrane systems to evaluate the binding free energies of the isoform complexes within a more biologically relevant context. Implicit membrane region incorporated into the solvation calculations and the program computed solvent excluded surfaces using both the water probe and the membrane probe. The last 20ns of MD trajectories were analyzed for binding free energy estimations. The MMPBSA calculated average binding free energies for E7EUV6, A0A2R8Y642, and C9J9N5 were -15.66, -31.00, and -32.05 kcal/mol, respectively (Figs. 3 and 4). Meanwhile canonical dimers for EAA1 and EAA2 remained at -17.63 and -29.56, respectively (Supplementary Figure S7, Table III). These results suggest a similar binding affinity for the truncated isoforms to the canonical transporters in membrane, which is an indicative of a competitive interaction against oligomerization. The standard variations of binding free energies for both isoform and canonical systems were noteworthy. Interestingly, isoform complexes showed slightly higher deviation of binding energies compared to canonical EAA2. Standard error of the mean (SEM) calculated from the propagation of uncertainty formula for A0A2R8Y642 and C9J9N5 was 3.44 and 4.04, respectively. For the canonical EAA2 dimer, it was reduced to 2.74 (Supplementary Figure S7). These deviations may result form the water penetration into the membrane interface, as SASA of the system was also slightly variable.

**Table III:** Interface Composition and Average Binding Free Energies (ΔΔGs) of the AlphaFold2 Multimer Predicted Complexes

Receptor	Ligand	Contact Composition^1^		Binding free energy (ΔΔG) (kcal/mol)^2^
		Receptor	Ligand	
EAA1 (Canonical)	EAA1 (Canonical)	ECL1, TM2, ECL2, TM5	ECL1, TM2, ECL2, TM4, ICL2, TM5	-17.63
EAA1 (Canonical)	E7EUV6	ECL2, TM4	TM2	-15.66
EAA2 (Canonical)	EAA2 (Canonical)	ECL2, TM4, ICL2	ECL1, TM2, ECL2, TM4, ICL2, TM5	-29.56
EAA2 (Canonical)	C9J9N5	ECL1, TM2, ECL2, TM5	ICL1, TM2, ICL2, TM4	-31.00
EAA2 (Canonical)	A0A2R8Y642	ECL2, TM4, ICL2	N-coil, TM1, ECL1, TM3, ICL2, TM4	-32.05
A0A2R8Y4N0	A0A2R8Y4N0	TM4, ICL2	TM4, ICL2	-29.31
H0Y7R2	H0Y7R2	TM4, ICL2	TM4, ICL2	-35.95

^1^Interface composition of the complex, domains contributing to interface surface with more than one residue are included

^2^Residue binding free energies are calculated according to MMPBSA algorithm through 30-50ns MD of the membrane systems (please see methods)

**Fig. 5:**
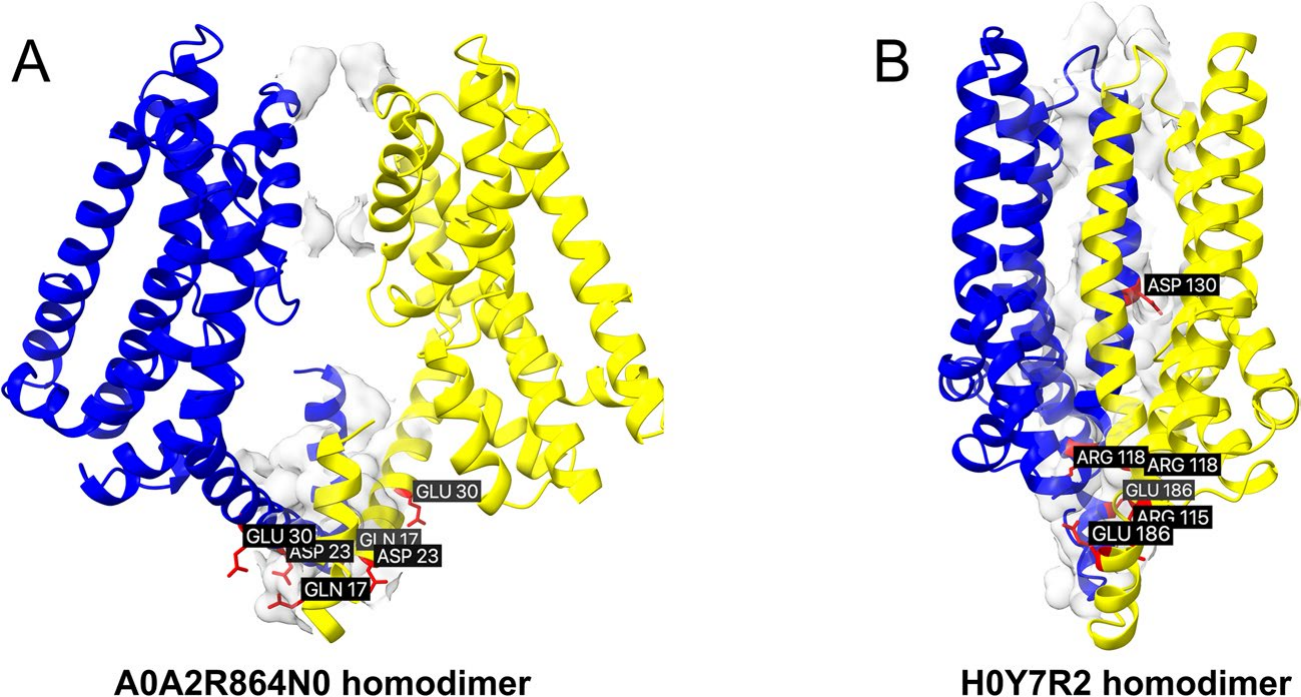
Self-assembly of A0A2R864N0 and H0Y7R2. Despite their lower ranking for canonical transporters, the isoforms are prone to self-assembly. The binding free energies of homodimers are calculated for: (**A**) A0A2R864N0 (-29.31 kcal/mol), and (**B**) H0Y7R2 (-35.95 kcal/mol). The interface surface is displayed, with the six (three for the ligand and three for the receptor) highest energy-contributing residues highlighted in red and labeled. Large loops are removed to facilitate direct comparisons.

The analysis of the A0A2R8Y4N0 isoforms revealed distinct self-binding properties that differentiate them from other isoforms of EAA2. The MMPBSA calculations indicated that the self-assembly tendency of the A0A2R8Y4N0 is significant, with a binding free energy of -29.31 kcal/mol (Table III, Fig. 5). For H0Y7R2 homodimer, it was -35.95 kcal/mol (Supplementary Figure S8, Fig. 5). The differences in binding to the canonical form between truncated EAA3 isoforms and EAA1 and EAA2 isoforms are likely a result of the structural changes brought by truncation. Interestingly, EAA3 transcripts were not only fewer in number but also has a stronger tendency to self-assembly.

### Residue-wise Decomposition Analysis

The binding interface involved residues at the distal or proximal ends of membrane domains (Supplementary Figures S9–S13). For EAA1 homodimer: 4 Arg, 1 Asp, 5 Glu, 2 Asn, and 3 Gln residues were contributing to binding free energy at least in ¾ of the timesteps (Supplementary Table S9). All of these amino acids are hydrophilic, with Arg, Asp, and Glu being charged. This unaltered interaction pattern indicates the stabilizing roles of these residues for the system. Despite the lack of contribution from Glu and Gln residues, the retained Arg, Asn, and Asp residues in the EAA1-E7EUV6 interaction appeared sufficient for binding (Supplementary Table S10). The heterodimer lacks contributions from several residues that were presented in the homodimer (e.g., Glu228, Gln261, Gln263). Hence, these residues would make special drug targets. Topologically, the highest energy contributing residue in both complexes was residue Arg173, which is located near the carboxyl end of T3 (or T3-T4 loop) that can be bonded with ligand TM residues. The arginine residues located near the core were involved in conformational changes and inhibition of the transporter [3, 30, 31]. Consequently, these interactions can be a determinant of the overall stability and modulation of the complex within the lipid bilayer. These findings have medical implications, GLUT1 Deficiency Syndrome is also due to mutations involved charged intracellular residues [32, 33].

The roles of Arg and Glu residues remained in EAA2 homodimer complex: 2 Arg, 3 Asp, 2 Glu, 2 Asn, 2 Gln, and 1 Lys residues were contributing to binding free energy at least in ¾ of the timesteps (Supplementary Table S11). Furthermore, only three residues (Arg, Asp, Glu) were apparent in ligand residues. For the EAA2-isoform complexes, two canonical residues (Asp83 and Arg48 for A0A2R8Y642; Asp34 and Asp 238 for C9J9N5) were further incorporated into the interaction, which explains the slightly stronger binding energy of isoforms. These residues were not seen in canonical EAA2 homodimer (Supplementary Tables S12, S13). Thus, they potentially make better targets for tailored inhibitor design.

Another important finding is the self-assembly tendencies of the truncated isoforms. The isoforms of A0A2R8Y4N0 and H0Y7R2 have very strong self-assembly tendencies to form homodimers (Fig. 5), mimicking canonical TM5 and TM6 helices have a supporting function. A0A2R8Y4N0 homodimer presents unique contributing residues including Hsd284, Tyr278, and several glycine residues (Supplementary Table S14). Meanwhile, H0Y7R2 homodimer shows significant contributions from threonine residues (Thr132, Thr158, Thr9) that were not seen in other isoforms (Supplementary Table S15). The isoform homodimers also had similar RMSF features while maintaining the gyration radius, indicating they are indeed as stable as canonical dimers (Supplementary Figure S14).

### Evolutionary Coupling of the Contact Surface

**Fig. 6:**
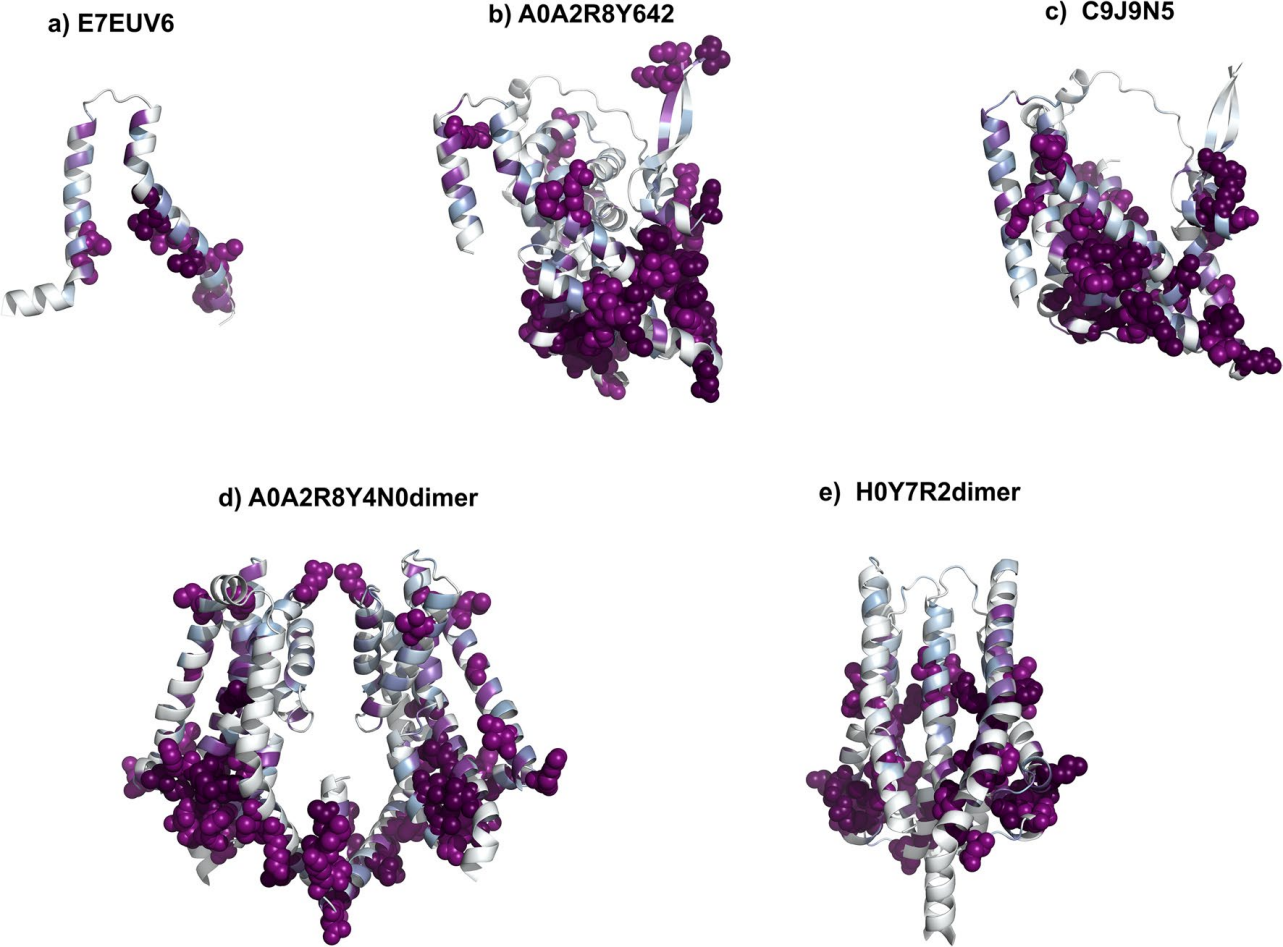
Residue co-evolution analysis of the truncated isoforms (**a**-**e**). A strong enrichment (colored purple) within the residues contributing to the binding interface (Figs. 3, 4 and 5) suggests that these regions are co-evolved.

The evolutionary coupling analysis was conducted to investigate co-evolutionary patterns among amino acid residues of truncated isoforms. Our results demonstrate a strong coupling within the residues of helices contributing to the binding of the truncated isoforms, indicating that these regions are co-evolved (Fig. 6). This suggests that the structural integrity and predicted interaction domains of these isoforms are preserved, which aligns with the hypothesis that these regions are crucial for protein–protein interactions. Interestingly, the analysis also revealed significant couplings within the intracellular residues of the EAA2 isoforms. This finding is particularly notable as it suggests potential interactions within the cellular environment that may play a role in intracellular signaling or structural organization. EAA2 and its isoforms are expressed in tissues where it frequently interacts with a variety of other proteins or cellular components (Table II). The discovery of the evolutionary couplings within the intracellular parts of truncated isoforms suggests these regions may have functional roles in regulatory mechanisms.

### Future Scopes and the Potential Applications

Our findings suggest that the studied isoforms, which exhibits structural similarities with trimerization domains, may interact with canonical glutamate transporters. The couplings of intracellular residues further show a potential for cellular interactions that could influence cellular processes. Conversely, self-binding of these isoforms could prevent undesired dimerization or trimerization with canonical transporters. Targeting the self-assembly process could disrupt isoform dimerization towards binding to canonical transporters, which would make distinct modulatory targets. These isoforms also have potential medical significance, and insights into the structural similarities and binding energy patterns of truncated isoforms could guide drug development. Involvement of charged Arg residues indicates additional drug-design applications. For EAA2, two canonical Asp residues were specifically involved in binding of truncated isoforms, which are potential tailored targets. The interactions with the lipid bilayer or membrane environment can influence the energetics of the system. The 50ns molecular dynamics simulations may be too short to capture all relevant conformational changes. The 50 ns duration should thus be viewed as providing initial insights into the interaction landscape of isoform-canonical dimers, rather than a comprehensive exploration of all possible states. To further validate and expand upon our findings, future studies should consider extended simulation times, particularly for key dimer configurations identified in this work. Additionally, enhanced sampling techniques such as meta-dynamics or umbrella sampling could be employed to more thoroughly explore the free energy landscape of these interactions. Despite these limitations, the consistent trends observed across our simulations provide a solid foundation for understanding the competitive binding of truncated isoforms to canonical glutamate transporters and guide future computational and experimental investigations. Longer molecular dynamics simulations and lipid distortion profiling could produce additional insights on membrane-specific conformational changes and their impact on protein–protein interactions [34]. Experimental studies would be beneficial for revealing exact effect of truncated isoforms on transport cycle.

The fact that pre-mRNA coding isoforms and canonical structures are expressed in distinct tissues suggests a level of specificity in the regulation of these transporters. This could have implications for the development of tissue-specific therapies or interventions targeting these transporters. Therefore, *in vivo* expression analysis of these isoforms could yield valuable insights. Identifying and monitoring these isoforms in patient samples may provide clinicians with valuable diagnostic information. Furthermore, understanding the genetic variations associated with these isoforms can provide indications for predicting susceptibility to certain conditions.

In summary, our comprehensive analysis of gene-centric isoform maps provides insights into the modulatory dynamics within glutamate transporters. From structural nuances and energetic landscapes, each analysis indicates an inhibitory potential of the truncated isoforms. Subsequent experimental validation could help corroborate these computational findings and predicted binding patterns.

## Methods

### Protein Sequence Alignments and other Characteristics

The protein sequence data was retrieved in FASTA format from the UniProt website https://www.uniprot.org [35]. For each canonical EAAT, their UniProt accession numbers are in () EAAT1 (P43003), EAAT2 (P43004), EAAT3 (P43005), EAAT4 (P48664) and EAAT 5 (O00341). The corresponding protein coding isoforms were listed utilizing Ensembl genome browser (Ensemble release 112) which identifies potential isoforms via automatic gene-centric mapping from eukaryotic reference proteomes [36]. These mappings are based on gene identifiers from Ensembl, EnsemblGenomes, model organism databases and original sequencing projects [36]. This study specifically analyses the protein-coding isoforms (not decayed) of the length of %85-%15 of the canonical full-length structures, as the nonsense-mediated mRNA decay (NMD) eliminates transcripts containing premature stop codons. The membrane topology and other sequence features then visualized by plots generated using Protter web application https://wlab.ethz.ch/protter/ [37]. The secondary structures and sequence alignments visualized using the 2dSS web server http://genome.lcqb.upmc.fr/2dss/ [38]. The website Expasy https://web.expasy.org/ was used to calculate the molecular weights (MW), amino acid composition, instability indexes and GRAVY indexes of the isoforms and canonical transporters [39–41]. Accordingly, hydrophilic isoforms (negative GRAVY scores) were excluded since they were outside the scope of this study. Namely, the isoforms H0YEB1, A0A2R8YHI4, E7EUS7, and A0A7P0T8Q1 were rejected.

### AlphaFold2 Predictions

AlphaFold2 Program (https://github.com/sokrypton/ColabFold) was used for the structure predictions of the studied isoforms following the instructions at the website on T4 GPUs with 16 GB of RAM that are available for all Colab users [42, 43]. Accordingly, Alphafold2_multimer_v3 was applied for complex predictions via the open source ColabFold pipeline; “templates” mode was not utilized to prevent the protocol becoming over-reliant on templates to sample starting positions of complexes, as it is recommended in such cases [28]. Input MSA features were generated by ColabFold using the “mmseqs2_uniref_env” MSA mode. By default, the MSAs pair mode were “unpaired & paired”, which utilizes both paired sequences from same species and unpaired MSA sequences. The European Bioinformatics Institute (EBI, https://alphafold.ebi.ac.uk) has all AlphaFold2-predicted structures. The Uniprot website (https://www.uniprot.org) has each protein ID, entry name, description, and FASTA sequence [35].

### Comparative Structural Analysis with Experimental Data

To evaluate the reliability of each predicted monomers, the AlphaFold2 prediction confidence profiles of the models summarized as well as the comparative analysis with available experimental structures. The experimentally-determined structures used in this study are EAA1 (PDB ID: 5LLU) [8], EAA2 (PDB ID: 7VR8, 7XR6) [30, 44], and EAA3 (PDB ID: 8CV2) [45] that were obtained from the RCSB PDB database [46] https://www.rcsb.org. Structural superposition with experimental structures were further quantified the reliability of the predicted models on par with the state-of-art methods of quality estimations [47]. The structures were superposed using PyMOL version 2 https://pymol.org/2/ [48], the similarities benchmarked quantitatively by the calculation of root mean square deviation (RMSD) values. Isoforms that could not be superposed with the canonical form (A0A7P0Z4R4, A0A7P0T9A4, and A0A7P0T807) were rejected from the sample.

To assess structural variability of the truncated isoforms, we used the canonical folded transporter monomers predicted by AlphaFold2 as the constant variables. While the output conformations of AlphaFold models cannot be reliably controlled, no conformational deviation that disrupts direct compressions was identified, all predictions corresponded to the outward-facing conformations. The deviations from the canonical structures were quantified by the root mean square deviation (RMSD) values. Since the truncated isoforms were analyzed according to helical similarities, the RMSD values calculated for helical domains (align, and ss H). The sample of structures subject to docking analysis included the isoforms of E7EUV6, A0A7P0T9Z4, C9J9N5, A0A2R8Y642, A0A2R8Y4N0, H0Y7R2, F1T0D4, and the canonical structures of EAAT1, EAAT2, EAAT3, EAAT4 and EAAT 5.

### Protein–protein Docking and Benchmarking

Alongside AlphaFold multimer v3, we utilized four conventional docking algorithms to identify protein–protein interactions: ClusPro default, ClusPro multimer, LightDock, and RosettaDock5 [49–55]. The trimerization option was supported only for ClusPro multimer and AlphaFold multimer. Hence, RosettaDock5, LightDock, and ClusPro default were utilized only for homodimer predictions. Each input contained AlphaFold2 predicted transporter subunits [42, 43]. The ClusPro server (cluspro.bu.edu) was run using default parameters and also in multimer mode [49, 50]. The LightDock server (server.lightdock.org) was run in flexible-backbone mode [51–53]. RosettaDock5 was run using the Rosetta Online Server That Includes Everyone (ROSIE), with default parameters [54, 55].

The ColabFold pipeline output contains all five models generated by AlphaFold multimer that were benchmarked in this study. For the resultant models of ClusPro, we selected top 10 models from all configurations: the default “Balanced,” the “Electrostatic-favored,” “Hydrophobic-favored,” and “VdW + Elec”. The recommended ranking within ClusPro server was utilized that considers cluster size [50]. For the resultant models of LightDock and RosettaDock, we included the top 10 predicted models in the output, which was ranked by the algorithms. The accuracy of these algorithms was analyzed by comparing the in silico generated models with experimentally determined dimer/trimer structures. The experimentally determined structures used in this study are EAA1 (PDB ID: 5LLU) [8], EAA2 (PDB ID: 7VR8, 7XR6) [30, 44], and EAA3 (PDB ID: 8CV2) [45], which were obtained from the RCSB PDB database [46] (https://www.rcsb.org). The resultant models were visualized using using PyMOL version 2 https://pymol.org/2/ and UCSF ChimeraX [48, 56]. The similarities were benchmarked quantitatively by superimposing structures and calculating root mean square deviation (RMSD) values using PyMOL [48]. Both all-atom RMSD and helical RMSD were calculated to assess the conformational similarities.

Since AlphaFold multimer models for canonical complexes were observed to be substantially more accurate than the remaining conventional docking algorithms, they are preferred for isoform-canonical docking throughout this study. However, the default ranking within AF Multimer was not reliable, with lower-ranked models sometimes exhibiting better binding affinity.

### Model Re-ranking

Considering its computational efficiency, MM/GBSA can be more suited to this study, where the correct ranking of inhibitors is more emphasized [57]. The Molecular Mechanics with Generalized Born and Surface Area (MM/GBSA) method implemented in the HawkDock web server http://cadd.zju.edu.cn/hawkdock was employed to re-rank the models [58]. Missing hydrogens and heavy atoms of the protein–protein complex was added by the tleap module in Amber16 [59]. The MM/GBSA is calculated based on the ff02 force field [60], and the GBOBC1 model [61]. The system was minimized for 5000 steps with a cutoff distance of 12Å for van der Waals interactions (2000 cycles of steepest descent and 3000 cycles of conjugate gradient minimizations) [58]. The key residues for both receptor (canonical transporter) and ligand (isoform) were highlighted by the free energy decomposition.

### Molecular Dynamics Simulations

Molecular dynamics simulations were conducted for the isoform-canonical dimer complexes with the best results from the molecular docking study, as determined by available experimental data and model re-ranking. All simulations and analyses were executed on Google Compute Engine, via an Ubuntu based Virtual Machine (VM) setup, utilizing a total of 236 core Intel Sapphire Rapid CPUs and 1648 GB RAM, 12 Local SSDs. Computational resources were selected based on the size and complexity of the molecular system with the lowest carbon impact, a grid carbon intensity of maximum 200 gCO2eq/kWh being ensured. To optimize computational efficiency, the simulations were parallelized across multiple processors or cores within VM, using MPI (Message Passing Interface) and OpenMP [62]. Throughout the simulation runs, resource utilization metrics such as CPU usage, memory consumption, and disk I/O were monitored. Configuration files and Linux bash codes for the simulations are publicly available with step-by-step instructions. Simulation input files, trajectory data, and analysis results were stored in the distributed file system provided by Google Compute Engine and accessed utilizing WinSCP 6.2, a SSH File Transfer Protocol (SFTP) client.

The membrane-protein systems were built using the membrane builder of the CHARMM-GUI web server [63–65]. The protein portion was centered in a rectangular box and protonation states assigned based on the local pH. Spatial positions of proteins with respect to the lipid bilayer are optimized by the PPM 2.0 method that accounts for the anisotropic water–lipid environment described by the dielectric constant and hydrogen-bonding profiles [66]. The membrane models generated consisted of 70% 1-palmitoyl-2-oleoyl-glycero-3-phosphocholine (POPC) and 30% cholesterol. The choice of this lipid composition is reasonable, as it represents a simplified model of the plasma membrane and in line with our previous publications [16, 17, 34]. The system was solvated in TIP3P water with 150 mM KCl. All MD simulations were performed using GROMACS 2022.3 [67]. The all-atom CHARMM36m [68] force field was used. The system energy was minimized using the steepest descent method and the maximum forces were converged below 1000 kJ/mol/nm. Electrostatics were treated with Particle Mesh Ewald (PME), and the cutoff for both Coulomb and van der Waals interactions was 1.2 nm. A multi-step minimization and equilibration process was used to relax the protein-membrane systems and to achieve stable equilibrium simulations. 125-ps equilibration simulations were performed using the standard six-step CHARMM-GUI protocol [65]. The Parrinello-Rahman barostat with semi-isotropic coupling and the Nose–Hoover thermostat was used. The temperature was held at 303.15 K and pressure was held at 1 bar, respectively. After NVT and NPT equilibration, a 50-ns production MD simulation was run with timestamps for each 500ps. The trajectories were later combined with gmx traj tool.

Trajectory analysis conducted for analyzing the stability of the systems. The root mean square deviation (RMSD) of the backbone atoms of the protein was calculated, with the gmx rms tool. The gyration radius of the protein atoms was calculated using the gmx gyrate tool. As a measure of the flexibility of residues, the RMSF was also calculated for the Cα atoms of proteins. The solvent accessible surface area (SASA) of the side chains of the protein residues was calculated by the gromacs gmx_sasa, with a solvent probe radius of 1.4 A [69]. The structures and 50ns trajectories were visualized using PyMOL https://pymol.org/2/ [48]. Frames on last 20ns (between 31 and 50ns) of equilibrated MD trajectories subjected to the MMPBSA binding free energy estimations.

### MMPBSA Calculations for Membrane-Protein Systems

The binding free energy of the complexes was calculated using Molecular Mechanics Poisson-Boltzmann Surface Area (MMPBSA) algorithm and performed using the gmx_MMPBSA tool [70, 71]. The parameter settings of MMPBSA calculation in membrane-bound protein systems followed the instruction of the Amber reference manual [70, 71]. To incorporate an implicit membrane region into the solvation calculations, we activated the membrane option by setting memopt to 1. The dielectric constant of the membrane was set to 7.0 (enem = 7.0); solute dielectric constant (indi), and the solvent dielectric constant (exdi) were set to 4 and 80, respectively. With the default options, the program computed solvent excluded surfaces using both the water probe (with a default prbrad of 1.40) and the membrane probe (with a default mprob of 2.70). The total electrostatic energy and forces were computed using the particle–particle particle-mesh (P3M) method described by Lu and Luo [72]. Consequently, the EPB term in the output file was set to zero, while the EEL term included both the reaction field energy (EPB) and the Coulombic energy (EEL). The van der Waals energy was calculated along with the particle–particle portion of the Coulombic energy. The summary file provides binding energies for each time step, the average binding energies and their standard deviations were further calculated. Standard error of the mean (SEM) was calculated using the propagation of uncertainty formula. Per-residue decomposition analysis was performed (idecomp = 2) to calculate residue contributions to the binding energy. This included adding 1–4 EEL terms to the total EEL and 1–4 VDW terms to the total VDW potential. Residues within 4Å of both the receptor and ligand were included in the output file. Binding surface compositions were further analyzed and visualized utilizing the gmx_MMPBSA_ana tool and UCSF ChimeraX [56]. Contact surfaces of isoform-canonical complexes were visualized according to residues within 4Å atomic distance. Plots that were generated through analyses were visualized utilizing Grace (https://plasma-gate.weizmann.ac.il/Grace/).

### Evolutionary Conservation Profiles and Coevolution of Binding Interfaces

The EVcouplings server (https://v2.evcouplings.org/) calculates ECs using a maximum entropy model [73], by the statistics of the multiple sequence alignments (MSAs). E-value thresholds can vary based on the length of the protein and the size of the database, making them incomparable across different proteins. Therefore, we utilized length-normalized bitscore thresholds as a more consistent measure of evolutionary distance. For large sequence alignments, Pseudo-likelihood maximization (PLM) becomes asymptotically equivalent to full maximum-likelihood, resulting in more accurate contact predictions [74]. Thus, in this work the recommended EVcouplings-PLM method was used to generate ECs of truncated isoforms.

## Supplementary Information

Below is the link to the electronic supplementary material.

Supplementary file1 (PDF 4.35 MB)

## Data Availability

Each statistical and computational analysis of this study, included with step-by-step instructions where possible, are publicly available to ensure repeatability. For more detailed information on the statistical analyses, input files and detailed outputs, including the AlphaFold2 calculations and codes to regenerate analyses, please visit the website: https://github.com/karagol-alper/EAAT-truncated-isoforms. Further information and requests for data should be directed to and will be fulfilled by S.Z., shuguang@media.mit.edu.
