# Supplementary material for: Inhibitory Potential of the Truncated Isoforms on Glutamate Transporter Oligomerization Identified by Computational Analysis of Gene-Centric Isoform Maps
Source: Pharm Res. 2024 Nov 1;41(11):2173–87. doi: 10.1007/s11095-024-03786-z (PMC11599315; doi:10.1007/s11095-024-03786-z)
Supplement: Supplementary file 1 — Supplementary file1 (PDF 4.35 MB) [file 11095_2024_3786_MOESM1_ESM.pdf]

Supplementary Information

Inhibitory potential of the truncated isoforms on glutamate transporter oligomerization identified by computational analysis of gene-centric isoform maps

Alper Karagöl[1,¶], Taner Karagöl[1,¶], Mengke Li[2], Shuguang Zhang[3,*]

[1]Istanbul University Istanbul Medical Faculty, Istanbul, Turkey

[2]State Key Laboratory of Microbial Metabolism, Joint International Research Laboratory of Metabolic and Developmental Sciences, and School of Life Sciences and Biotechnology, Shanghai Jiao Tong University, Shanghai 200240, China

[3]Laboratory of Molecular Architecture, Media Lab, Massachusetts Institute of Technology, 77 Massachusetts Avenue, Cambridge, MA, 02139, USA

¶These authors contribute equally.

*To whom the correspondence should be addressed.

Email:

Alper Karagöl, alper.karagol@gmail.com ORCID: 0009-0001-7864-0732

Taner Karagöl, taner.karagol@gmail.com ORCID: 0009-0005-1011-7661

Shuguang Zhang, Shuguang@MIT.EDU ORCID: 0000-0002-3856-3752

Table of Contents

**1.**	Figure S1. Flowchart of methodology adopted for the proposed computational approach.	Pg. 3
**2.**	Table S1. Structural features of the sampled EAATs isoforms.	Pg. 4
**3.**	Figure S2: Sequence Alignments and Canonical Topology of Glutamate Transporter Isoforms.	Pg. 5-7
**4.**	Table S2. Comparison of experimental complexes with models predicted utilizing different algorithms.	Pg. 8-10
**5.**	Table S3. MMGBSA re-ranking of the isoform-canonical dimers.	Pg. 11-12
**6.**	Table S4. Interface composition and single-point MMGBSA predictions (ΔΔGs) of the sampled dimer complexes.	Pg. 13
**7.**	Table S5. MMGBSA re-ranking of the isoform homodimers.	Pg. 14-15
**8.**	Table S6. Interface composition and single-point MMGBSA predictions (ΔΔGs) of the AlphaFold predicted isoform homodimers.	Pg. 16
**9.**	Table S7. MMGBSA re-ranking of the isoform-canonical trimers.	Pg. 17
**10.**	Table S8. Interface composition and single-point MMGBSA predictions (ΔΔGs) of the sampled trimer complexes.	Pg. 18
**11.**	Figure S3. Residue-wise stability analysis of the EAA2 isoform complexes and EAA2 homodimer after 50ns MD.	Pg. 19
**12.**	Figure S4. Residue-wise stability analysis of the E7EUS6 isoform complexes and EAA1 homodimer after 50ns MD.	Pg. 20
**13.**	Figure S5. Temporal Radius of Gyration of EAA2 isoform complexes and EAA2 homodimer through 50ns MD.	Pg. 21
**14.**	Figure S6. The temporal evolution of solvent accessible surface areas (SASAs) of the selected complexes.	Pg. 21
**15.**	Figure S7. MMPBSA binding energy calculations of isoform-canonical complexes through 30-50ns MD simulation.	Pg. 22
**16.**	Figure S8. MMPBSA binding energy calculations of isoform self-assembly complexes through 30-50ns MD simulation.	Pg. 23
**17.**	Figure S9. Residue contributions to the binding free energy of the canonical EAA1-EAA1 complex.	Pg. 24
**18.**	Figure S10. Residue contributions to the binding free energy of the E7EUV6-EAA1 complex.	Pg. 25
**19.**	Figure S11. Residue contributions to the binding free energy of the A0A2R8Y642-EAA2 complex.	Pg. 26
**20.**	Figure S12. Residue contributions to the binding free energy of the canonical C9J9N5-EAA2 complex.	Pg. 27
**21.**	Figure S13. Residue contributions to the binding free energy of the canonical EAA2-EAA2 complex.	Pg. 28
**22.**	Table S9. Residue-wise decomposition of MMPBSA calculations of canonical EAA1 homodimer through 40 timestep MD (20ns)	Pg. 29
**23.**	Table S10. Residue-wise decomposition comparison of MMPBSA calculations of EAA1-E7EUV6 dimer through 40 timestep MD (last 20ns).	Pg. 30
**24.**	Table S11. Residue-wise decomposition of MMPBSA calculations of canonical EAA2 dimer through 40 timestep MD (last 20ns).	Pg. 31
**25.**	Table S12. Residue-wise decomposition comparison of MMPBSA calculations of EAA2_A0A2R8Y642 dimer through 40 timestep MD (last 20ns).	Pg. 32
**26.**	Table S13. Residue-wise decomposition comparison of MMPBSA calculations of EAA2_A0A2R8Y642 dimer through 40 timestep MD (last 20ns).	Pg. 33
**27.**	Table S14. Residue-wise decomposition of MMPBSA calculations of A0A2R8Y4N0 homodimer through 40 timestep MD (last 20ns).	Pg. 34
**28.**	Table S15. Residue-wise decomposition of MMPBSA calculations of H0Y7R2 homodimer through 40 timestep MD (last 20ns).	Pg. 35-36
**29.**	Figure S14. Residue-wise stability analysis of the sampled isoform dimers, A0A2R8YDN0 (a) and H0Y7R2 (b).	Pg. 37

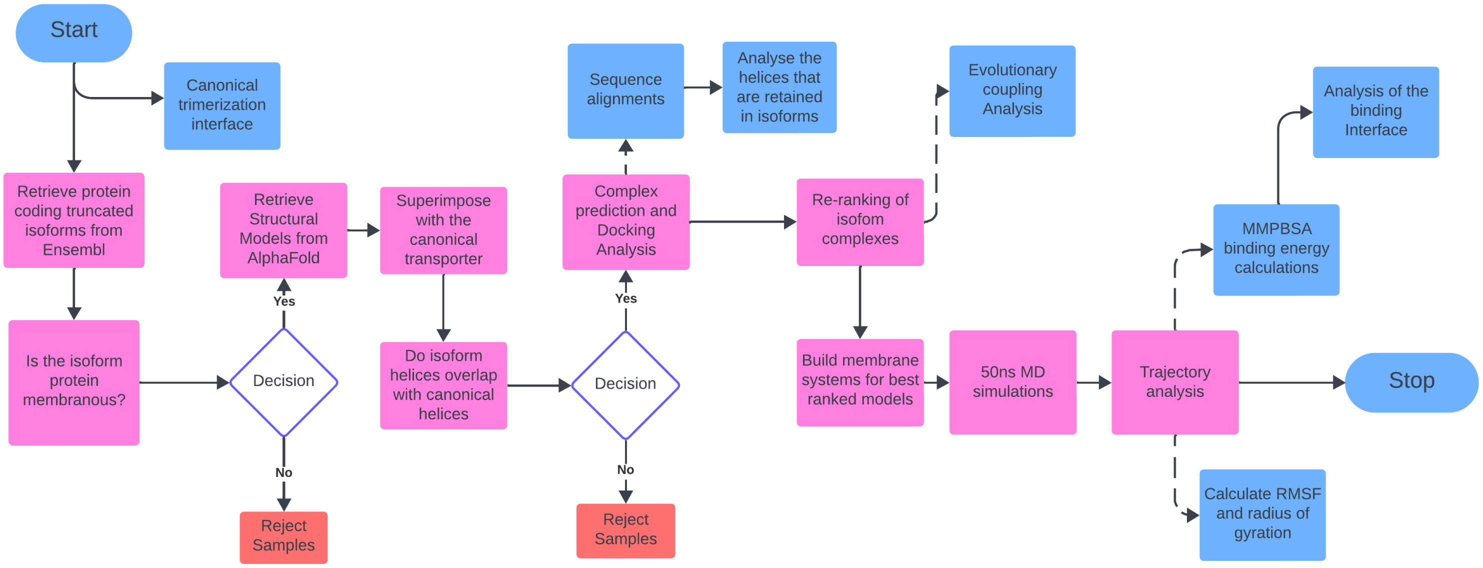

Figure S1. Flowchart of methodology adopted for the proposed computational approach. Sample deduction methodology starting from the computationally mapped isoform sequences, for identifying truncated isoforms that have modulatory effects on trimerization. For more information, please see Methods.

Table S1. Structural features of the sampled EAATs isoforms.

Name	Isoform ID[1]	Length[2]	Mass (Da)	TM count[3]	TM composition	RMSD (helical)	Note
EAA1 (SLC1 A3)	P43003 (Canonical)	542	59572	8	Canonical	0Å	
	E7EUV6	106	12250	2	TM1, TM2	3.069Å	
	A0A7P0T9Z4	123	12792	3	TM6, HP1, HP2	3.927Å	
	A0A7P0T9A4	162	17758	2	-	17.692Å	Low RMSD of helical residues
	A0A7P0T807	148	16348	3	-	13.176Å	Low RMSD of helical residues
	A0A087X0U3	430	47100	5	-	8.273Å	Low RMSD of helical residues
EAA2 (SLC1 A2)	P43004 (Canonical)	574	62104	8	Canonical	0Å	
	C9J9N5	409	44319	7	TM1, TM2, TM3, TM4, TM5, TM6, HP1	0.448Å	
	A0A2R8Y4D1	397	43126	7	TM1, TM2, TM3, TM4, TM5, TM6, HP1	4.202Å	Identical TM composition to C9J9N5, Lower RMSD
	A0A2R8Y642	435	46890	4	TM2, TM3, TM4, TM5	0.468Å	
	A0A2R8Y4N0	330	35816	5	TM5, TM6, HP1, TM7, HP2	0.590Å	
EAA3 (SLC1 A1)	P43005 (Canonical)	524	57100	8	Canonical	0Å	
	H0Y7R2	242	26753	2	TM5, TM6	3.228Å	
EAA4 (SLC1 A6)	P48664 (Canonical)	564	61565	8	Canonical	0Å	No sampled isoforms
EAA5 (SLC1 A7)	O00341 (Canonical)	560	60,658	8	Canonical	0Å	
	F1T0D4	472	51244	7	TM1, TM4, TM5, TM6, HP1, TM7, HP2	0.665Å	

[1]The Uniprot entry ID of the isoform (only included isoforms that have length between 15% and 85% of the canonical sequence) (Methods).
[2]The amino acid length of the isoform sequence.
[3]Transmembrane (TM) domain count of the protein, derived from the topology information included in the Uniprot entries.

Figure S2: Sequence Alignments and Canonical Topology of Glutamate Transporter Isoforms (a-c). Schematic representation of the canonical topology of glutamate transporters. Transmembrane helices (TM1-TM8, blue) and extracellular regions (pinkish), intracellular regions (yellow) and peripheral domains or hairpin loops (green) are illustrated. Below the topology illustrations is the sequence alignment of glutamate transporter isoforms, highlighting conserved and variable regions. Inserted residues or varied domains that were not in canonical sequence are highlighted in red.

a, EAA1

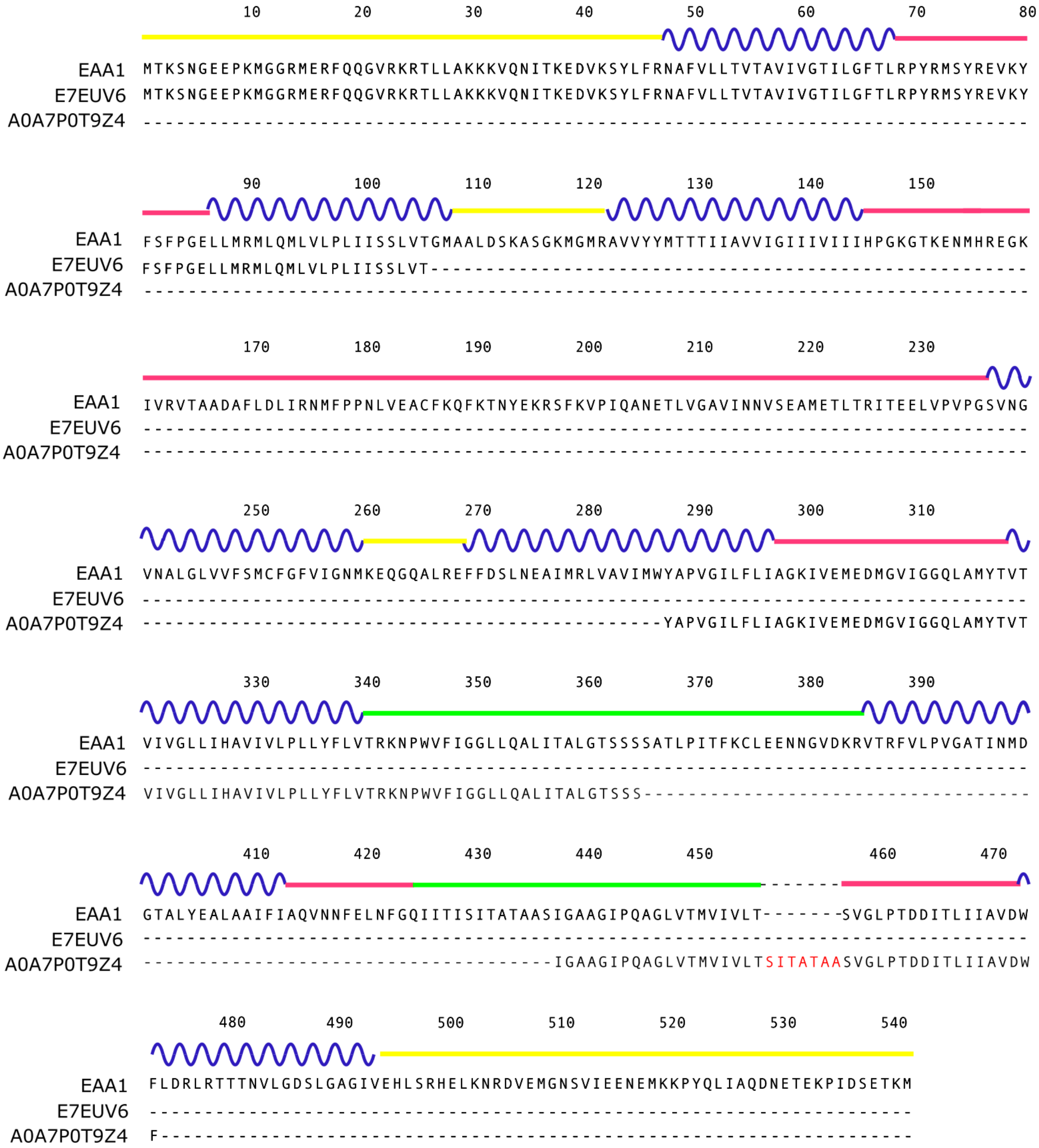

b, EAA2

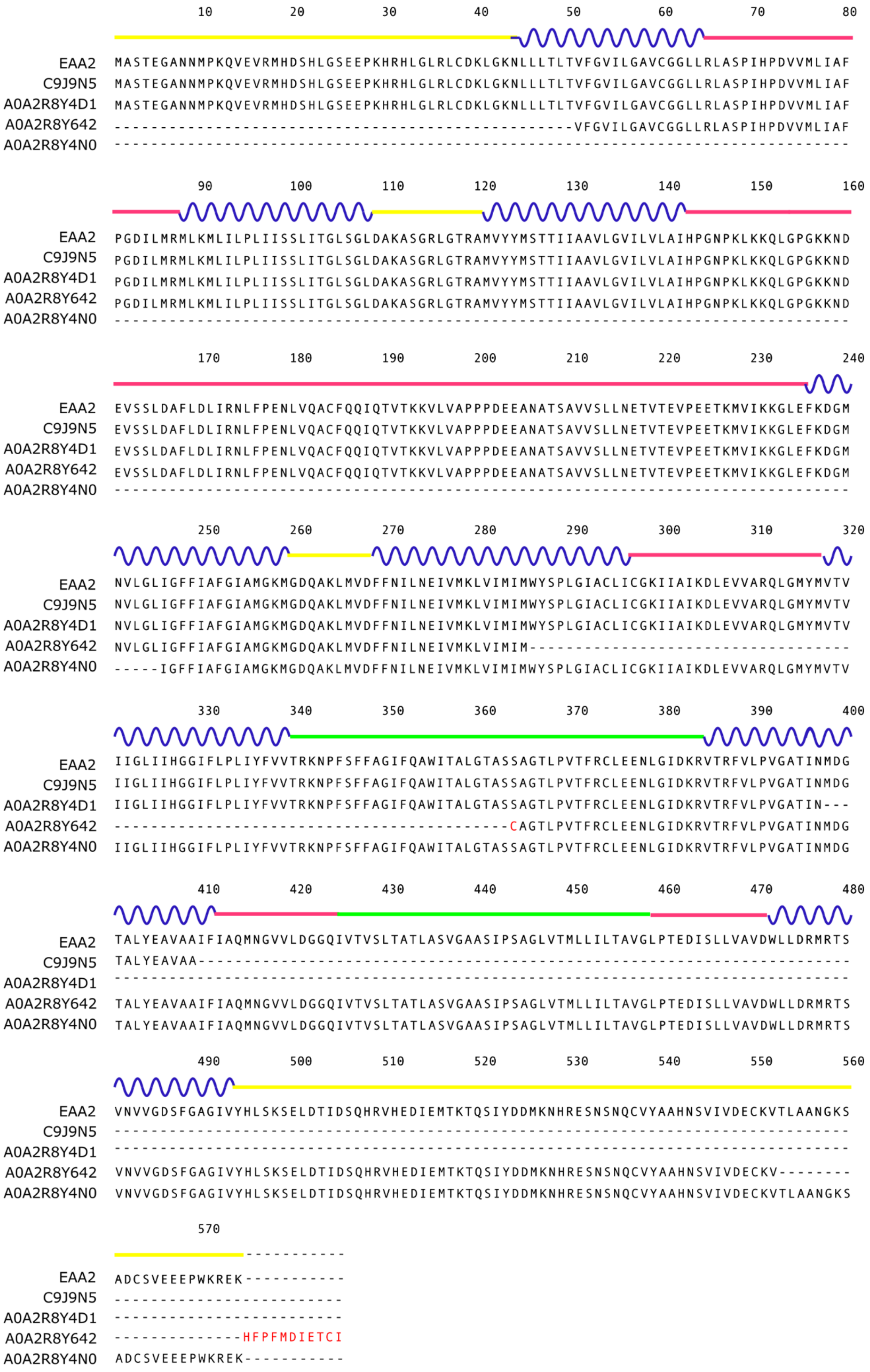

10 20 30 40 50 60 70 80
EAA2 MASTEGANNMPKQVEVRMHDSHLGSEEPKHRHLGLRLCDKLGKNLLLTLTVFGVILGAVCGGLLRLASPIHPDVVMLIAF
C9J9N5 MASTEGANNMPKQVEVRMHDSHLGSEEPKHRHLGLRLCDKLGKNLLLTLTVFGVILGAVCGGLLRLASPIHPDVVMLIAF
A0A2R8Y4D1 MASTEGANNMPKQVEVRMHDSHLGSEEPKHRHLGLRLCDKLGKNLLLTLTVFGVILGAVCGGLLRLASPIHPDVVMLIAF
A0A2R8Y642 --VFGVILGAVCGGLLRLASPIHPDVVMLIAF
A0A2R8Y4N0 --
90 100 110 120 130 140 150 160
EAA2 PGDILMRMLKMLILPLIISSLITGLSGLDAKASGRLGTRAMVYYMSTTIIAAVLGVILVLAIHPGNPKLKKQLGPGKKND
C9J9N5 PGDILMRMLKMLILPLIISSLITGLSGLDAKASGRLGTRAMVYYMSTTIIAAVLGVILVLAIHPGNPKLKKQLGPGKKND
A0A2R8Y4D1 PGDILMRMLKMLILPLIISSLITGLSGLDAKASGRLGTRAMVYYMSTTIIAAVLGVILVLAIHPGNPKLKKQLGPGKKND
A0A2R8Y642 PGDILMRMLKMLILPLIISSLITGLSGLDAKASGRLGTRAMVYYMSTTIIAAVLGVILVLAIHPGNPKLKKQLGPGKKND
A0A2R8Y4N0 --
170 180 190 200 210 220 230 240
EAA2 EVSSLDAFLDLIRNLFPENLVQACFQQIQTVTKKVLVAPPPDEEANATSAVVSLLNETVTEVPEETKMVIKKGLEFKDGM
C9J9N5 EVSSLDAFLDLIRNLFPENLVQACFQQIQTVTKKVLVAPPPDEEANATSAVVSLLNETVTEVPEETKMVIKKGLEFKDGM
A0A2R8Y4D1 EVSSLDAFLDLIRNLFPENLVQACFQQIQTVTKKVLVAPPPDEEANATSAVVSLLNETVTEVPEETKMVIKKGLEFKDGM
A0A2R8Y642 EVSSLDAFLDLIRNLFPENLVQACFQQIQTVTKKVLVAPPPDEEANATSAVVSLLNETVTEVPEETKMVIKKGLEFKDGM
A0A2R8Y4N0 --
250 260 270 280 290 300 310 320
EAA2 NVLGLIGFFIAFGIAMGKMGDQAKLMVDFFNILNEIVMKLVIMIMWYSPLGIACLICGKIIAIKDLEVVARQLGMYMVTV
C9J9N5 NVLGLIGFFIAFGIAMGKMGDQAKLMVDFFNILNEIVMKLVIMIMWYSPLGIACLICGKIIAIKDLEVVARQLGMYMVTV
A0A2R8Y4D1 NVLGLIGFFIAFGIAMGKMGDQAKLMVDFFNILNEIVMKLVIMIMWYSPLGIACLICGKIIAIKDLEVVARQLGMYMVTV
A0A2R8Y642 NVLGLIGFFIAFGIAMGKMGDQAKLMVDFFNILNEIVMKLVIMIM-----------------------------------
A0A2R8Y4N0 -----IGFFIAFGIAMGKMGDQAKLMVDFFNILNEIVMKLVIMIMWYSPLGIACLICGKIIAIKDLEVVARQLGMYMVTV
330 340 350 360 370 380 390 400
EAA2 IIGLIIHGGIFLPLIYFVVTRKNPFSFFAGIFQAWITALGTASSAGTLPVTFRCLEENLGIDKRVTRFVLPVGATINMDG
C9J9N5 IIGLIIHGGIFLPLIYFVVTRKNPFSFFAGIFQAWITALGTASSAGTLPVTFRCLEENLGIDKRVTRFVLPVGATINMDG
A0A2R8Y4D1 IIGLIIHGGIFLPLIYFVVTRKNPFSFFAGIFQAWITALGTASSAGTLPVTFRCLEENLGIDKRVTRFVLPVGATIN---
A0A2R8Y642 ---CAGTLPVTFRCLEENLGIDKRVTRFVLPVGATINMDG
A0A2R8Y4N0 IIGLIIHGGIFLPLIYFVVTRKNPFSFFAGIFQAWITALGTASSAGTLPVTFRCLEENLGIDKRVTRFVLPVGATINMDG
410 420 430 440 450 460 470 480
EAA2 TALYEAVAAIFIAQMNGVVLDGGQIVTVSLTATLASVGAASIPSAGLVTMLLILTAVGLPTEDISLLVAVDWLLDRMRTS
C9J9N5 TALYEAVAA---
A0A2R8Y4D1 --
A0A2R8Y642 TALYEAVAAIFIAQMNGVVLDGGQIVTVSLTATLASVGAASIPSAGLVTMLLILTAVGLPTEDISLLVAVDWLLDRMRTS
A0A2R8Y4N0 TALYEAVAAIFIAQMNGVVLDGGQIVTVSLTATLASVGAASIPSAGLVTMLLILTAVGLPTEDISLLVAVDWLLDRMRTS
490 500 510 520 530 540 550 560
EAA2 VNVVGDSFGAGIVYHLSKSELDTIDSQHRVHEDIEMTKTQSIYDDMKNHRESNSNQCVYAAHNSVIVDECKVTLAANGKS
C9J9N5 --
A0A2R8Y4D1 --
A0A2R8Y642 VNVVGDSFGAGIVYHLSKSELDTIDSQHRVHEDIEMTKTQSIYDDMKNHRESNSNQCVYAAHNSVIVDECKV--------
A0A2R8Y4N0 VNVVGDSFGAGIVYHLSKSELDTIDSQHRVHEDIEMTKTQSIYDDMKNHRESNSNQCVYAAHNSVIVDECKVTLAANGKS
570
EAA2 ADCSVEEEPWKREK-----------
C9J9N5 -------------------------
A0A2R8Y4D1 -------------------------
A0A2R8Y642 --------------HFPFMDIETCI
A0A2R8Y4N0 ADCSVEEEPWKREK-----------

c, EAA3

```
               10        20        30        40        50        60        70        80
EAA3   MGKPARKGCEWKRFLKNNWVLLSTVAAVVLGITTGVLVREHSNLSTLEKFYFAFPGEILMRMLKLIILPLIISSMITGVA
H0Y7R2 --------------------------------------------------------------------------------

               90       100       110       120       130       140       150       160
EAA3   ALDSNVSGKIGLRAVVYYFCTTLIAVILGIVLVVSIKPGVTQKVGEIARTGSTPEVSTVDAMLDLIRNMFPENLVQACFQ
H0Y7R2 --------------------------------------------------------------------------------

              170       180       190       200       210       220       230       240
EAA3   QYKTKREEVKPPSDPEMNMTEESFTAVMTTAISKNKTKEYKIVGMYSDGINVLGLIVFCLVFGLVIGKMGEKGQILVDFF
H0Y7R2 ------------------------------------------------------------------------------FF

              250       260       270       280       290       300       310       320
EAA3   NALSDATMKIVQIIMCYMPLGILFLIAGKIIEVEDWEIFRKLGLYMATVLTGLAIHSIVILPLIYFIVVRKNPFRFAMGM
H0Y7R2 NALSDATMKIVQIIMCYMPLGILFLIAGKIIEVEDWEIFRKLGLYMATVLTGLAIHSIVILPLIYFIVVRKNPFRFAMGM

              330       340       350       360       370       380       390       400
EAA3   AQALLTALMISSSSATLPVTFRCAEENNQVDKRITRFVLPVGATINMDGTALYEAVAAVFIAQLNDLDLGIGQIITISIT
H0Y7R2 AQALLTALMISSSSATLPVTFRCAEENNQVDKRITRFVLPVGATINMDGTALYEAVAAVFIAQLNDLDLGIGQIITIR--

              410       420       430       440       450       460       470       480
EAA3   ATSASIGAAGVPQAGLVTMVIVLSAVGLPAEDVTLIIAVDWLLDRFRTMVNVLGDAFGTGIVEKLSKKELEQMDVSSEVN
H0Y7R2 -------------------------------------------DRFRTMVNVLGDAFGTGIVEKLSKKELEQMDVSSEVN

              490       500       510       520
EAA3   IVNPFALESTILDNEDSDTKKSYVNGGFAVDKSDTISFTQTSQF
H0Y7R2 IVNPFALESTILDNEDSDTKKSYVNGGFAVDKSDTISFTQTSQF
```

Table S2. Comparison of experimental complexes with models predicted utilizing different algorithms.

Name	Stoichiometry	Model ID	RMSD (excluding outliers)	RMSD
**EAA1**	Homotrimer	Alphafold-Multimerv3_modelrank002	1.703 (69)	3.11
		Alphafold-Multimerv3_modelrank001	1.946 (43)	5.88
		Alphafold-Multimerv3_modelrank005	9.336 (18)	9.91
		Alphafold-Multimerv3_modelrank004	9.552 (24)	10.23
		Alphafold-Multimerv3_modelrank003	33.117 (0)	33.117
		ClusProMultimer(beta)-000.06	31.304 (6)	31.66
		ClusProMultimer(beta)-000.08	31.298 (3)	31.52
		ClusProMultimer(beta)-002.04	31.304 (6)	31.66
		ClusProMultimer(beta)-002.08	31.147 (3)	31.37
		ClusProMultimer(beta)-006.05	34.250 (2)	34.44
	Homodimer	Alphafold-Multimerv3_modelrank002	1.532 (46)	2.97
		Alphafold-Multimerv3_modelrank003	1.930 (53)	4.32
		Alphafold-Multimerv3_modelrank005	9.171 (19)	10.38
		Alphafold-Multimerv3_modelrank004	9.178 (14)	9.64
		Alphafold-Multimerv3_modelrank001	18.20 (3)	17.733
		RostettaDockv5-r0090	9.556 (12)	10.47
		RostettaDockv5-r0672	9.562 (12)	10.48
		RostettaDockv5-r0092	9.569 (12)	10.48
		RostettaDockv5-r0365	9.552 (13)	10.49
		RostettaDockv5-r0492	9.592 (12)	10.51
		ClusProMultimer(beta)-000.01	15.905 (35)	18.29
		ClusProMultimer(beta)- 002.01	15.897 (28)	17.14
		ClusProMultimer(beta)- 004.01	15.669 (27)	16.86
		ClusProMultimer(beta)- 006.01	12.426 (12)	13.06
		ClusProMultimer(beta)- 02.006.03	7.990 (147)	14.44
		ClusProDock-000.07	13.361 (63)	16.33
		ClusProDock-002.09	13.690 (70)	17.01
		ClusProDock-002.07	17.878 (47)	20.67
		ClusProDock-004.07	18.406 (78)	23.80
		ClusProDock-000.04	18.470 (78)	23.88
		LightDock-mintop1	25.416 (28)	27.68
		LightDock-mintop4	27.696 (10)	28.58
		LightDock-mintop7	27.980 (12)	29.07
		LightDock-mintop8	28.230 (12)	29.19
		LightDock-mintop9	29.161 (3)	29.45
**EAA2**	Homotrimer	Alphafold-Multimerv3_modelrank005	1.499 (243)	2.82
		Alphafold-Multimerv3_modelrank004	1.554 (190)	3.98
		Alphafold-Multimerv3_modelrank003	33.671 (0)	33.671
		Alphafold-Multimerv3_modelrank001	33.790 (3)	33.99

		Alphafold-Multimerv3_modelrank002	9.898 (38)	10.58
		ClusProMultimer(beta)-006.06	29.268 (12)	29.87
		ClusProMultimer(beta)-000.08	29.920 (0)	29.920
		ClusProMultimer(beta)-004.08	29.985 (8)	30.58
		ClusProMultimer(beta)-004.04	30.311 (5)	30.55
		ClusProMultimer(beta)-002.02	30.238 (0)	30.238
	Homodimer	Alphafold-Multimerv3_modelrank004	1.545 (132)	2.67
		Alphafold-Multimerv3_modelrank002	10.262 (15)	11.05
		Alphafold-Multimerv3_modelrank001	10.403 (12)	10.75
		Alphafold-Multimerv3_modelrank003	10.466 (10)	10.74
		Alphafold-Multimerv3_modelrank005	16.739 (1)	16.78
		RostettaDockv5-r0457	10.224 (16)	10.66
		RostettaDockv5-r0234	10.267 (14)	10.65
		RostettaDockv5-r0898	10.251 (15)	10.66
		RostettaDockv5-r0764	10.258 (15)	10.67
		RostettaDockv5-r0224	10.209 (17)	10.68
		ClusProMultimer(beta)-000.02	14.963 (3)	15.09
		ClusProMultimer(beta)-002.03	14.634 (2)	14.75
		ClusProMultimer(beta)-000.08	14.781 (1)	14.86
		ClusProMultimer(beta)-002.04	14.963 (3)	15.09
		ClusProMultimer(beta)-000.09	16.892 (1)	16.94
		ClusProDock- 000.09	15.090 (3)	15.22
		ClusProDock-002.06	15.139 (3)	15.27
		ClusProDock-000.03	20.884 (23)	22.28
		ClusProDock-002.04	20.884 (23)	22.28
		ClusProDock-004.04	21.040 (21)	22.34
		LightDock-mintop1	18.348 (1)	18.40
		LightDock-mintop4	20.628 (24)	22.09
		LightDock- mintop5	20.762 (44)	23.75
		LightDock- mintop8	19.015 (1)	19.12
		LightDock-mintop9	19.220 (1)	19.33
**EAA3**	Homotrimer	Alphafold-Multimerv3_modelrank002	1.540(15)	1.67
		Alphafold-Multimerv3_modelrank003	1.332(21)	2.06
		Alphafold-Multimerv3_modelrank001	1.208(41)	2.16
		Alphafold-Multimerv3_modelrank005	34.302 (3)	34.47
		Alphafold-Multimerv3_modelrank004	35.403 (0)	35.403
		ClusProMultimer(beta)-000.08	14.973 (0)	14.973
		ClusProMultimer(beta)-002.09	18.623 (18)	19.19
		ClusProMultimer(beta)-004.03	20.751 (24)	21.62
		ClusProMultimer(beta)-002.06	22.556 (12)	22.99
		ClusProMultimer(beta)-000.00	22.975 (13)	23.46
	Homodimer	Alphafold-Multimerv3_modelrank003	1.296(19)	1.42
		Alphafold-Multimerv3_modelrank005	8.158(28)	8.80
		Alphafold-Multimerv3_modelrank001	15.319 (1)	15.36

		Alphafold-Multimerv3_modelrank002	15.378(1)	15.42
		Alphafold-Multimerv3_modelrank004	15.360(1)	15.40
		RostettaDockv5-r0357	15.252 (0)	15.252
		RostettaDockv5-r0298	15.258 (0)	15.258
		RostettaDockv5-r0300	15.268 (0)	15.268
		RostettaDockv5-r0404	15.281 (0)	15.281
		RostettaDockv5-r0824	15.281 (0)	15.281
		ClusProMultimer(beta)-002.07	9.660 (0)	9.660
		ClusProMultimer(beta)-002.01	12.929 (15)	13.48
		ClusProMultimer(beta)-000.01	12.929 (15)	13.48
		ClusProMultimer(beta)-002.05	11.547 (82)	15.84
		ClusProMultimer(beta)-000.06	11.547 (82)	15.84
		ClusProDock-000.05	10.940 (165)	22.76
		ClusProDock-002.03	10.976 (126)	17.85
		ClusProDock-004.03	10.976 (126)	17.85
		ClusProDock-000.02	10.976 (126)	17.85
		ClusProDock-004.04	11.914 (155)	22.51
		LightDock-mintop4	14.915 (16)	15.62
		LightDock-mintop10	19.792 (9)	20.30
		LightDock- mintop9	20.761 (52)	24.48
		LightDock- mintop5	24.164 (25)	26.00
		LightDock-mintop3	27.959 (2)	28.13

Table S3. MMGBSA re-ranking of the isoform-canonical dimers.

Name	Result ID	VDW	ELE	GB	SA	MMGBSA (kcal/mol)
**EAA1**	EAA1_E7EUV6AFrank1-1701081904949	-93.01	-127.81	157.43	-12.77	-76.17
	EAA1_E7EUV6AFrank2-1701081920081	-88.74	-138.08	169.04	-12.47	-70.26
	EAA1_E7EUV6AFrank3-1701081937176	-94.44	-122.92	151.25	-13.06	-79.16
	EAA1_E7EUV6AFrank4-1701081948152	-93	-148.73	179.63	-13	-75.1
	EAA1_E7EUV6AFrank5-1701081963140	-91.44	-155.06	183.53	-12.65	-75.62
	EAA1_A0A7P0T9Z4rank1-1718629074717	-4.88	-13.20	23.64	-0.06	5.50
	EAA1_A0A7P0T9Z4rank2-1718629090241	-5.99	20.61	-8.85	-0.31	5.45
	EAA1_A0A7P0T9Z4rank3-1718629106203	-8.54	-4.41	18.85	-0.54	5.36
	EAA1_A0A7P0T9Z4rank4-1718629119879	-11.90	25.56	-7.12	-1.13	5.42
	EAA1_A0A7P0T9Z4rank5-1718629137801	-10.52	-21.41	37.99	-0.71	5.35
**EAA2**	EAA2_A0A2R8Y642AFrank1-1701094847733	-162.63	-355.63	429.61	-20.13	-108.78
	EAA2_A0A2R8Y642AFrank2-1701094860976	-167.26	-277.51	357.31	-20.56	-108.02
	EAA2_A0A2R8Y642AFrank3-1701094874576	-161.84	-337.84	409.63	-20.13	-110.19
	EAA2_A0A2R8Y642AFrank4-1701094902545	-161.1	-270.71	342.18	-20.07	-109.69
	EAA2_A0A2R8Y642AFrank5-1701094918394	-163.78	-358.48	425.91	-20.75	-117.09
	EAA2_C9J9N5AFrank1-1701095009054	-158.89	14.49	61.58	-19.82	-102.64
	EAA2_C9J9N5AFrank2-1701095039385	-160.68	0.21	74.68	-20	-105.78
	EAA2_C9J9N5AFrank3-1701095057983	-160.25	6.56	72.89	-19.66	-100.46

	EAA2_C9J9N5AFrank4-1701095071905	-158.45	16.73	59.99	-19.69	-101.43
	EAA2_C9J9N5AFrank5-1701095083305	-155.52	17.19	52.08	-19.46	-105.72
	EAA2_A0A2R8Y4N0AFrank1-1701094752292	-63.49	7.95	27.98	-7.29	-34.85
	EAA2_A0A2R8Y4N0AFrank2-1701094766372	-64.27	10.18	25.38	-7.36	-36.08
	EAA2_A0A2R8Y4N0AFrank3-1701094784001	-64.77	14.65	26.58	-7.6	-31.14
	EAA2_A0A2R8Y4N0AFrank4-1701094800099	-63.66	-83.4	119.51	-7.37	-34.93
	EAA2_A0A2R8Y4N0AFrank5-1701094815515	-65.63	-80.8	115.54	-7.57	-38.46
**EAA3**	EAA3_H0Y7R2AFrank1-1701149227294	-48.89	94.66	-59.41	-5.72	-19.36
	EAA3_H0Y7R2AFrank2-1701149252104	-49.06	131.53	-94.29	-5.9	-17.71
	EAA3_H0Y7R2AFrank3-1701149269342	-48.7	57.91	-21.83	-5.87	-18.49
	EAA3_H0Y7R2AFrank4-1701149280505	-49.66	69.71	-35.02	-5.72	-20.69
	EAA3_H0Y7R2AFrank5-1701149291632	-49.6	125.98	-89.9	-5.79	-19.31
**EAA5**	EAA5_F1T0D4AFrank1-1701151625200	-153.73	-26.16	105.72	-19.56	-93.73
	EAA5_F1T0D4AFrank2-1701151632003	-203.5	-167.86	277.86	-25.18	-118.68
	EAA5_F1T0D4AFrank3-1701151650670	-208.55	-552.59	660.83	-26.38	-126.69
	EAA5_F1T0D4AFrank4-1701151644212	-237.75	-37	158.91	-29.17	-145.02
	EAA5_F1T0D4AFrank5-1701151660253	-210.71	-156.89	277.29	-27.94	-118.25

Table S4. Interface composition and single-point MMGBSA predictions (ΔΔGs) of the sampled dimer complexes.

Receptor	Ligand[1]	Model ID	Contact Composition[2]		MMGBSA (kcal/mol)[3]
			Receptor	Ligand	
**EAA1**	P43003 (Canonical)	X-ray (dimer)	ECL2, TM4, ICL2	ECL1, TM2, ECL2, TM4, ICL2, TM5	-100.85
	P43003 (Canonical)	AlphaFold2 (dimer)	ECL2, TM4, ICL2	ECL1, TM2, ECL2, TM4, ICL2, TM5	-110.52
	E7EUV6	AlphaFold2 (dimer)	ECL2, TM4	TM2	-79.16
**EAA2**	P43004 (Canonical)	EM (dimer)	ECL2, TM4, ICL2	ECL1, TM2, ECL2, TM4, ICL2, TM5	-110.13
	P43004 (Canonical)	AlphaFold2 (dimer)	ECL1, TM2, ECL2, TM5	ECL1, TM2, ECL2, TM4, ICL2, TM5	-102.26
	C9J9N5	AlphaFold2 (dimer)	ECL1, TM2, ECL2, TM5	ICL2, TM4	-105.78
	A0A2R8Y642	AlphaFold2 (dimer)	ECL1, TM2, ECL2, TM5	ECL1, TM3	-110.19
	A0A2R8Y4N0	AlphaFold2 (dimer)	TM4, ICL2	N-coil, TM5	-38.46
**EAA3**	P43005 (Canonical)	EM (dimer)	ECL1, TM2, ECL2, ICL2, TM5	ECL2, TM4, ICL2	-108.37
	P43005 (Canonical)	AlphaFold2 (dimer)	ECL1, TM2, ECL2, TM4, ICL2, TM5	ECL2, TM4, ICL2	-107.05
	H0Y7R2	AlphaFold2 (dimer)	TM4, ICL2	N-coil, TM1	-20.69

[1]The Uniprot Entry ID of the isoform, canonical structures of the corresponding isoforms are stated in parentheses.
[2]Interface composition of the complex.
[3]Residue binding free energies are calculated according to MMGBSA algorithm.

Table S5. MMGBSA re-ranking of the isoform homodimers.

Name	Result ID	VDW	ELE	GB	SA	MMGBSA (kcal/mol)
**E7EUV6**	E7EUV6dimerAFrank1-1701348017658	-37.28	758.12	-718.12	-4.72	-2.01
	E7EUV6dimerAFrank2-1701348027913	-46.97	690.15	-649.80	-5.68	-12.30
	E7EUV6dimerAFrank3-1701348040665	-22.98	696.69	-662.94	-2.79	7.99
	E7EUV6dimerAFrank4-1701348057049	-37.28	758.12	-718.12	-4.72	-2.01
	E7EUV6dimerAFrank5-1701348068850	-52.68	542.79	-510.80	-6.59	-27.29
**A0A7P0T9Z4**	A0A7P0T9Z4dimerrank2-1718628959856	-90.42	110.50	-50.79	-11.28	-41.99
	A0A7P0T9Z4dimerrank4-1718628994842	-67.43	227.74	-184.36	-7.89	-31.95
	A0A7P0T9Z4dimerrank5-1718629014290	-71.22	283.68	-241.10	-8.19	-36.83
**A0A2R8Y642**	A0A2R8Y642dimerAFrank1-1701359591331	-199.65	754.63	-646.17	-24.91	-116.11
	EAA2_A0A2R8Y642AFrank2-1701094860976	-154.04	432.95	-357.88	-19.49	-98.45
	EAA2_A0A2R8Y642AFrank3-1701094874576	-161.96	698.33	-603.34	-20.10	-87.07
	EAA2_A0A2R8Y642AFrank4-1701094902545	-158.90	668.87	-581.57	-20.38	-91.98
	EAA2_A0A2R8Y642AFrank5-1701094918394	-161.02	678.41	-584.27	-20.50	-87.38
**C9J9N5**	C9J9N5dimerAFrank1-1701359275426	-152.72	56.68	12.23	-19.16	-102.97
	C9J9N5dimerAFrank2-1701359289983	-154.48	27.93	44.21	-19.56	-101.89
	C9J9N5dimerAFrank3-1701359301339	-150.50	-27.64	95.05	-18.98	-102.07
	C9J9N5dimerAFrank4-1701359315191	-151.07	-21.71	85.46	-18.81	-106.13
	C9J9N5dimerAFrank5-1701359325661	-149.23	21.39	42.25	-18.69	-104.28

**A0A2R8Y4N0**	A0A2R8Y4N0dimerAFrank1-1701368544734	-105.91	399.41	-343.31	-13.72	-63.53
	A0A2R8Y4N0dimerAFrank2-1701368562423	-95.85	504.02	-452.71	-12.03	-56.57
	A0A2R8Y4N0dimerAFrank3-1701368577561	-136.36	604.56	-530.96	-17.72	-80.48
	A0A2R8Y4N0dimerAFrank4-1701368594635	-112.84	657.17	-592.53	-14.43	-62.63
	A0A2R8Y4N0dimerAFrank5-1701368606609	-215.56	623.18	-505.81	-28.45	-126.63
**H0Y7R2**	H0Y7R2dimerAFrank1-1701368720301	-183.97	196.24	-89.69	-26.76	-104.17
	H0Y7R2dimerAFrank3-1701368746691	-187.01	220.47	-97.70	-24.49	-88.74
	H0Y7R2dimerAFrank4-1701368758886	-199.31	209.82	-85.19	-26.47	-101.14
	H0Y7R2dimerAFrank5-1701368782757	-207.59	245.15	-129.27	-26.60	-118.30
**F1T0D4**	F1T0D4dimerAFrank2-1701359929594	-107.89	94.16	-3.46	-13.33	-30.51
	F1T0D4dimerAFrank3-1701359942028	-104.28	76.02	7.72	-12.67	-33.21
	F1T0D4dimerAFrank4-1701359954139	-149.94	51.58	53.64	-18.35	-63.07
	F1T0D4dimerAFrank5-1701359986871	-132.60	86.90	14.99	-16.90	-47.61

Table S6. Interface composition and single-point MMGBSA predictions (ΔΔGs) of the AlphaFold predicted isoform homodimers.

Receptor[1]	Ligand	Contact Composition[2]		MMGBSA (kcal/mol)[3]
		Receptor	Ligand	
**E7EUV6 (EAA1)**	E7EUV6	TM1, ICL1	TM1, ICL1	-27.29
**C9J9N5 (EAA2)**	C9J9N5	ICL1, TM5, ECL2	ICL2, TM4, ECL2	-106.13
**A0A2R8Y642 (EAA2)**	A0A2R8Y642	ECL1, TM3, ICL2	N-loop, TM4	-116.11
**A0A2R8Y4N0 (EAA2)**	A0A2R8Y4N0	N-loop, TM1, TM3, TM5, C-loop	N-loop, TM1, TM3, TM5, C-loop	-126.63
**H0Y7R2 (EAA3)**	H0Y7R2	N-loop, TM1, C-loop	N-loop, TM1, C-loop	-118.3

[1]The Uniprot Entry ID of the isoform, canonical structures of the corresponding isoforms are stated in parentheses.
[2]Interface composition of the complex.
[3]Residue binding free energies are calculated according to MMGBSA algorithm.

Table S7. MMGBSA re-ranking of the isoform-canonical trimers.

Name	Result ID	VDW	ELE	GB	SA	MMGBSA (kcal/mol)
**EAA1**	EAA1dimer_E7EUV6rank1-1701368904784	-78.51	-0.84	34.76	-10.97	-55.56
	EAA1dimer_E7EUV6rank2-1701368918683	-80.23	-11.88	49.54	-11.03	-53.60
	EAA1dimer_E7EUV6rank3-1701368935884	-82.72	-53.85	89.90	-11.31	-57.99
	EAA1dimer_E7EUV6rank4-1701368948688	-81.67	-25.36	66.99	-11.09	-51.13
	EAA1dimer_E7EUV6rank5-1701368960616	-76.66	-75.38	104.71	-10.84	-58.18
**EAA2**	EAA2dimer_A0A2R8Y642AFrank1-1719575937015	-314.92	-727.32	857.50	-39.26	-224.00
	EAA2dimer_A0A2R8Y642AFrank2-1719576046757	-323.88	-501.52	654.75	-39.87	-210.52
	EAA2dimer_A0A2R8Y642AFrank3-1719576058857	-319.35	-688.28	831.19	-40.02	-216.45
	EAA2dimer_A0A2R8Y642AFrank4-1719576069226	-310.82	-590.09	726.00	-38.92	-213.82
	EAA2dimer_A0A2R8Y642AFrank5-1719576100744	-327.26	-830.12	970.67	-41.01	-227.72
	EAA2dimer_C9J9N5AFrank1-1701767973766	-314.46	-81.95	224.64	-39.48	-211.25
	EAA2dimer_C9J9N5AFrank2-1701767990490	-335.94	-25.57	169.92	-43.20	-234.79
	EAA2dimer_C9J9N5AFrank3-1701768029822	-317.97	24.34	126.85	-38.89	-205.67
	EAA2dimer_C9J9N5AFrank4-1701768181329	-312.28	-70.54	207.20	-38.80	-214.42
	EAA2dimer_C9J9N5AFrank5-1701768196691	-315.60	28.12	120.71	-38.61	-205.38

Table S8. Interface composition and single-point MMGBSA predictions (ΔΔGs) of the sampled trimer complexes.

Receptor	Ligand[1]	Model ID	Contact Composition[2]		MMGBSA (kcal/mol)[3]
			Receptor	Ligand	
**EAA1**	P43003 (Canonical)	X-ray (trimer)	A-ECL1, TM2, ECL2, TM5<br>B- ECL2, TM4, ICL2	ECL1, TM2, ECL2, TM4, ICL2, TM5	-196.65
	P43003 (Canonical)	AlphaFold2 (trimer)	A-ECL1, TM2, ECL2, TM5<br>B- ECL2, TM4, ICL2	ECL1, TM2, ECL2, TM4, ICL2, TM5	-212.94
	E7EUV6	AlphaFold2 (trimer)	A-ECL2, TM4<br>B-	TM2	-58.18
**EAA2**	P43004 (Canonical)	EM (trimer)	A- ECL2, TM4, ICL2<br>B- ECL1, TM2, ECL2, TM5	ECL1, TM2, ECL2, TM4, ICL2, TM5	-221.13
	P43004 (Canonical)	AlphaFold2 (trimer)	A- ECL2, TM4, ICL2<br>B- ECL1, TM2, ECL2, TM5	ECL1, TM2, ECL2, TM4, ICL2, TM5	-212.45
	C9J9N5	AlphaFold2 (trimer)	A- ECL1, TM2, ECL2, TM5<br>B- ECL2, TM4, ICL2	ICL1, TM2, ICL2, TM4	-234.79
	A0A2R8Y642	AlphaFold2 (trimer)	A-ECL2, TM4, ICL2<br>B- ECL1, TM2, ECL2, TM5	N-coil, TM1, ECL1, TM3, ICL2, TM4	-227.72
	A0A2R8Y4N0	AlphaFold2 (trimer)	A-TM4, ICL2<br>B- ICL2, TM5	N-coil, TM1	-92.91
**EAA3**	P43005 (Canonical)	EM (trimer)	A- ECL2, TM4, ICL2<br>B- ECL1, TM2, ECL2, ICL2, TM5	ECL1, TM2, ECL2, TM4, ICL2, TM5	-216.91
	P43005 (Canonical)	AlphaFold2 (trimer)	A- ECL2, TM4, ICL2<br>B- ECL1, TM2, ECL2, TM4, ICL2, TM5	ECL1, TM2, ECL2, TM4, ICL2, TM5	-216.71
	H0Y7R2	AlphaFold2 (trimer)	A-<br>B- TM4, ICL2	N-coil, TM1	-23.05

[1]The Uniprot Entry ID of the isoform, canonical structures of the corresponding isoforms are stated in parentheses.
[2]Interface composition of the complex.
[3]Residue binding free energies are calculated according to MMGBSA algorithm.

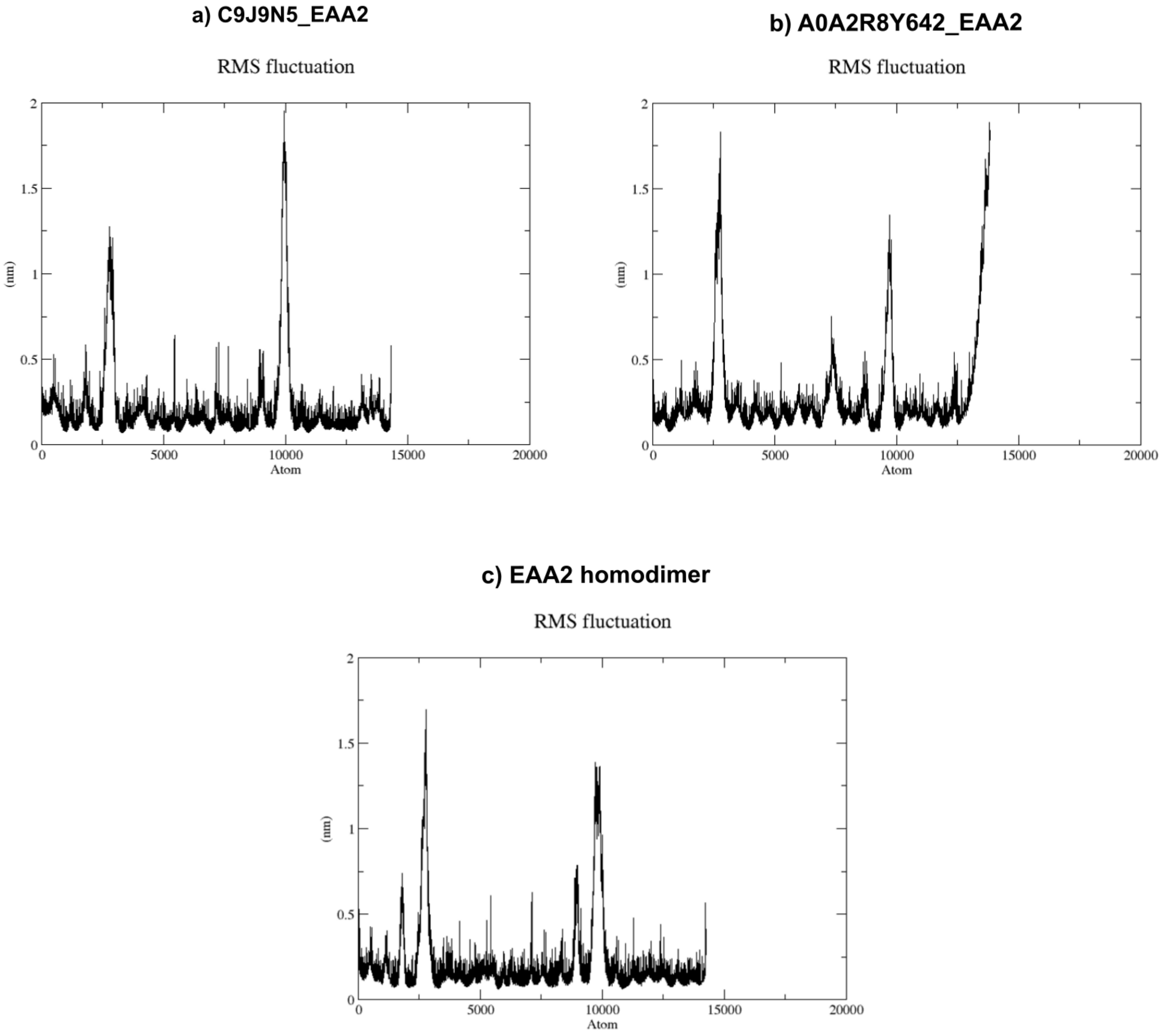

Figure S3. Residue-wise stability analysis of the EAA2 isoform complexes and EAA2 homodimer after 50ns MD. Spikes corresponds to flexible loops (Specially, N-termini, ECL1, ECL2, C-termini), isoform systems have comparable stability to canonical EAA2 homodimer.

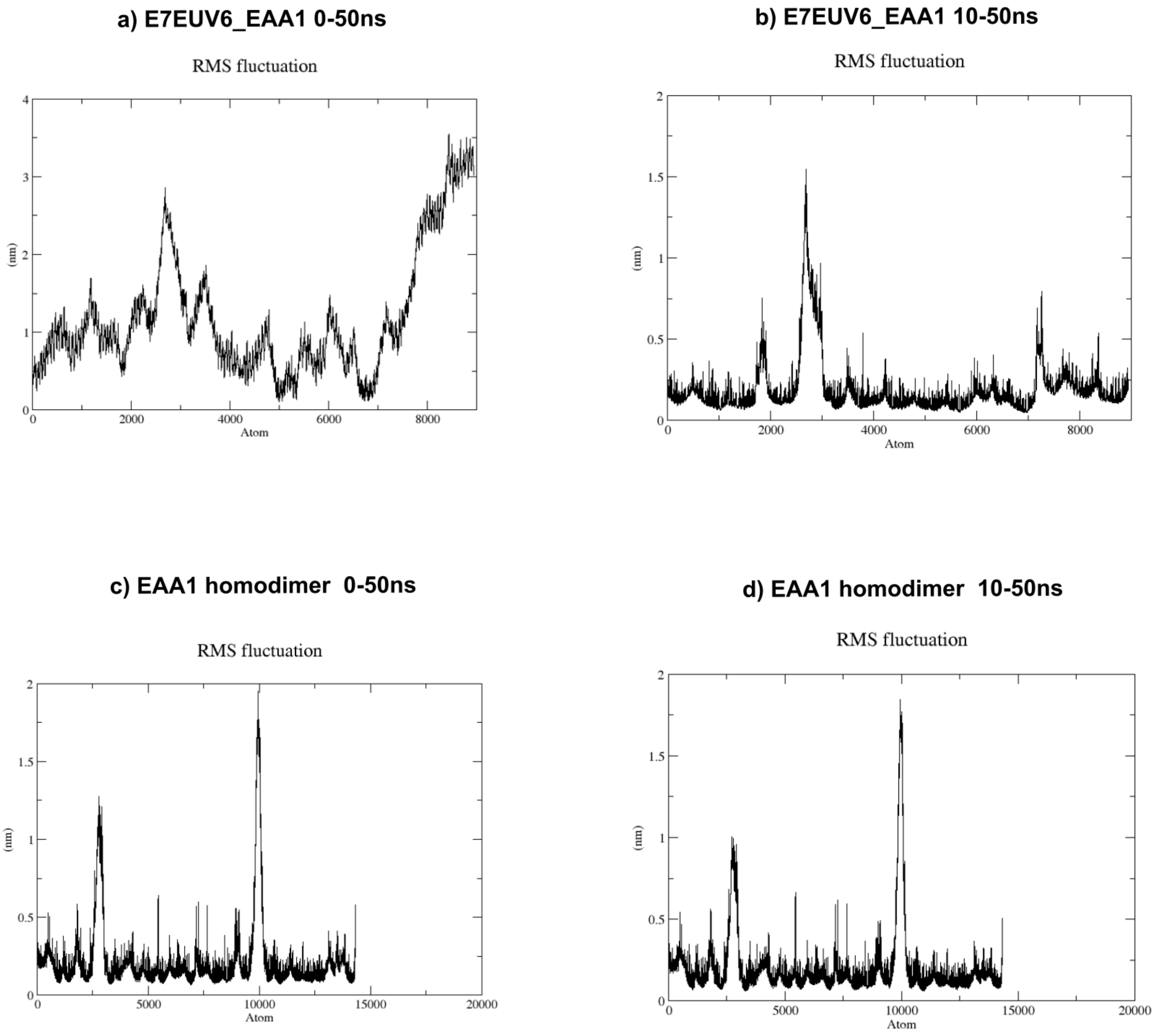

Figure S4. Residue-wise stability analysis of the E7EUS6 isoform complexes and EAA1 homodimer after 50ns MD. Spikes corresponds to flexible loops (Specially, N-termini, ECL1, ECL2, C-termini), E7EUS6 system stabilize after 10ns MD, became to have a comparable stability to canonical EAA1 homodimer.

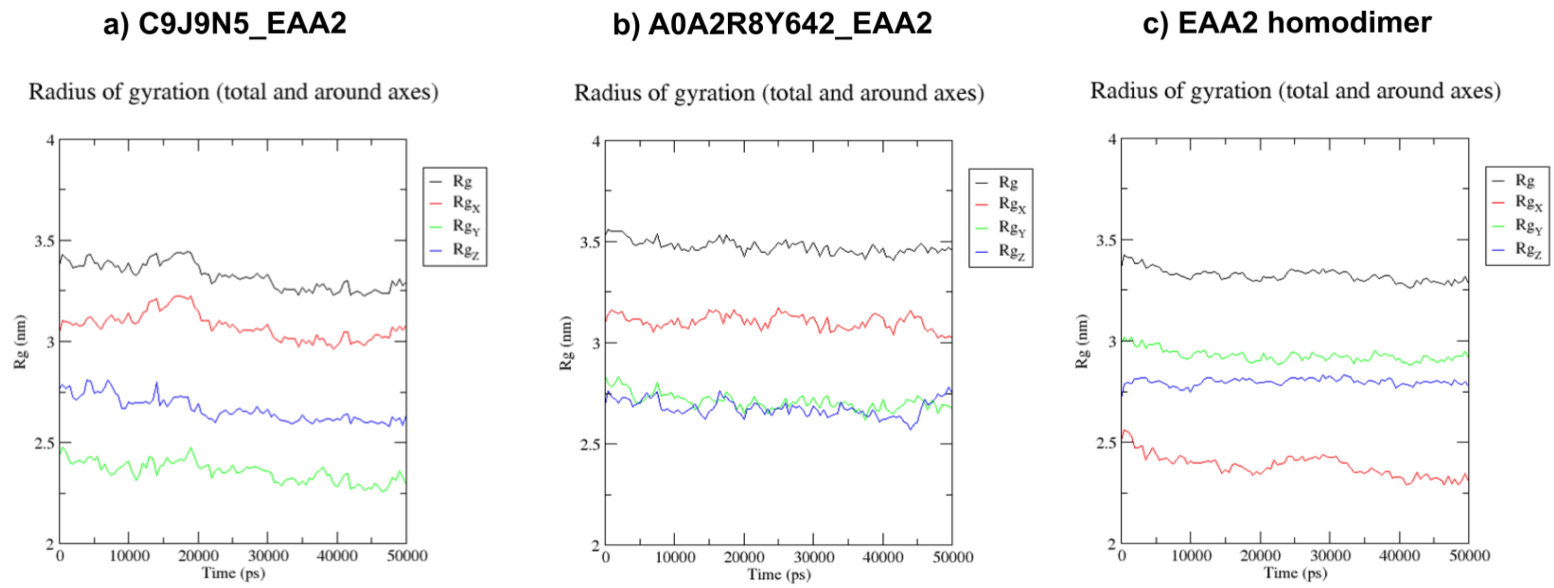

Figure S5. Temporal Radius of Gyration of EAA2 isoform complexes and EAA2 homodimer through 50ns MD.

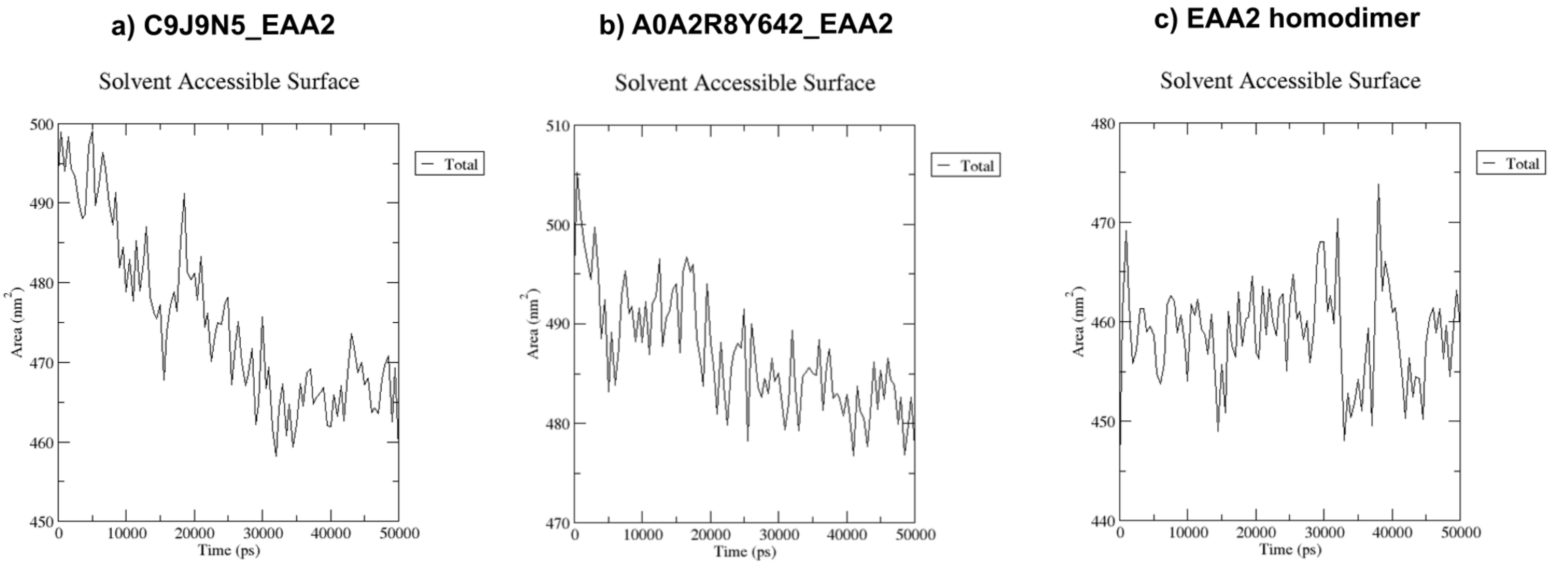

Figure S6. The temporal evolution of solvent accessible surface areas (SASAs) of the selected complexes.

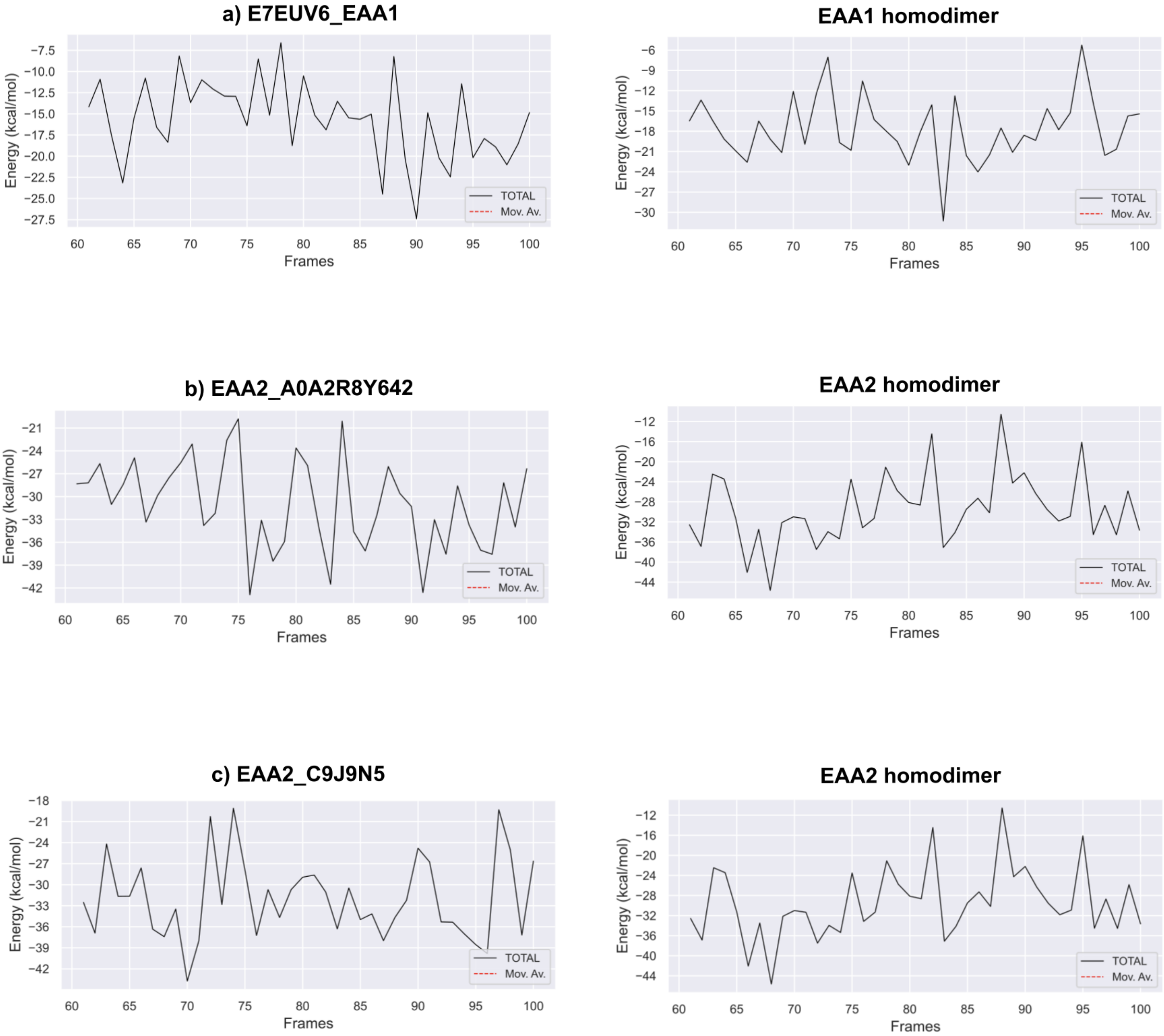

Figure S7. MMPBSA binding energy calculations of isoform-canonical complexes through 30-50ns MD simulation.

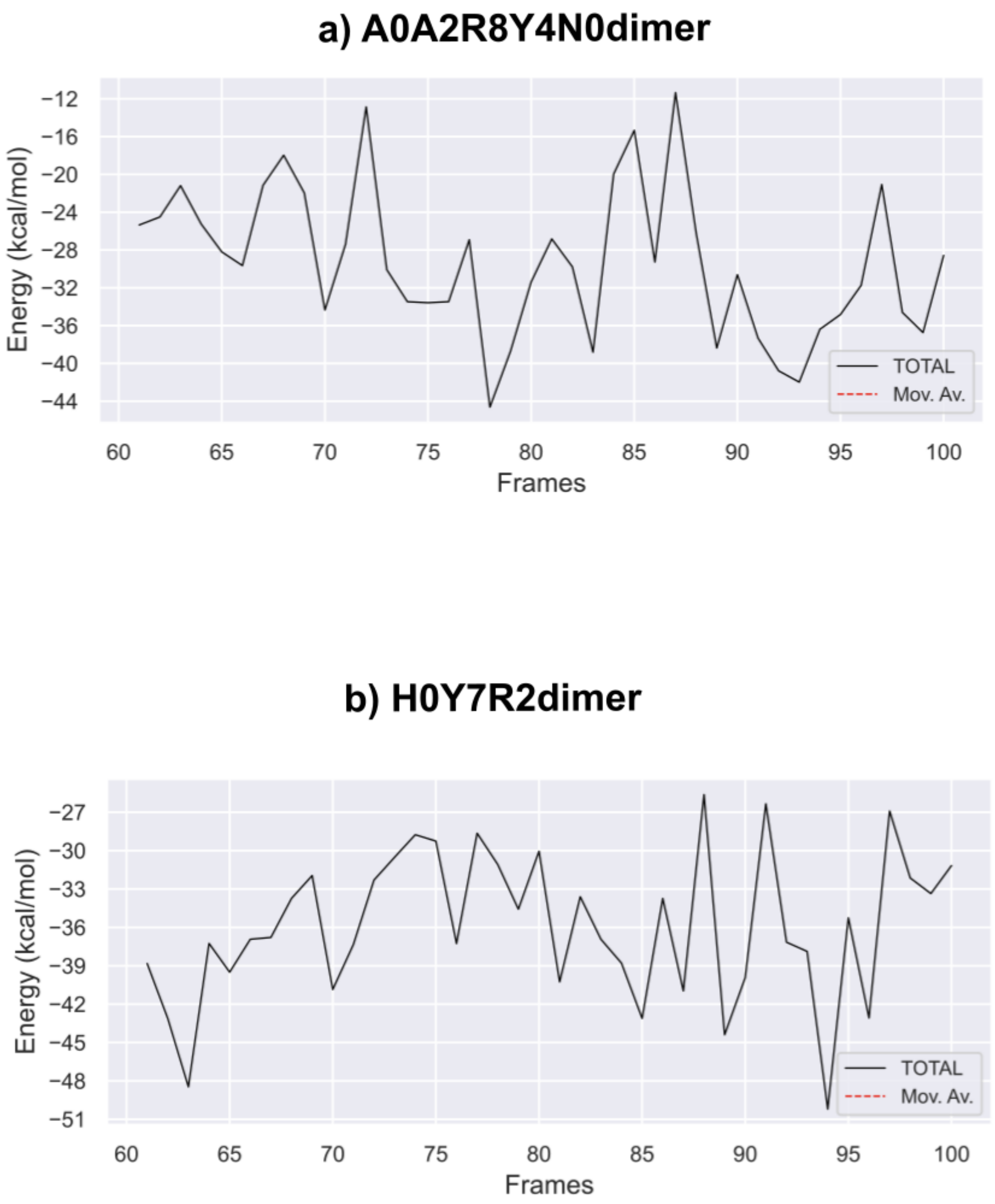

Figure S8. MMPBSA binding energy calculations of isoform self-assembly complexes through 30-50ns MD simulation.

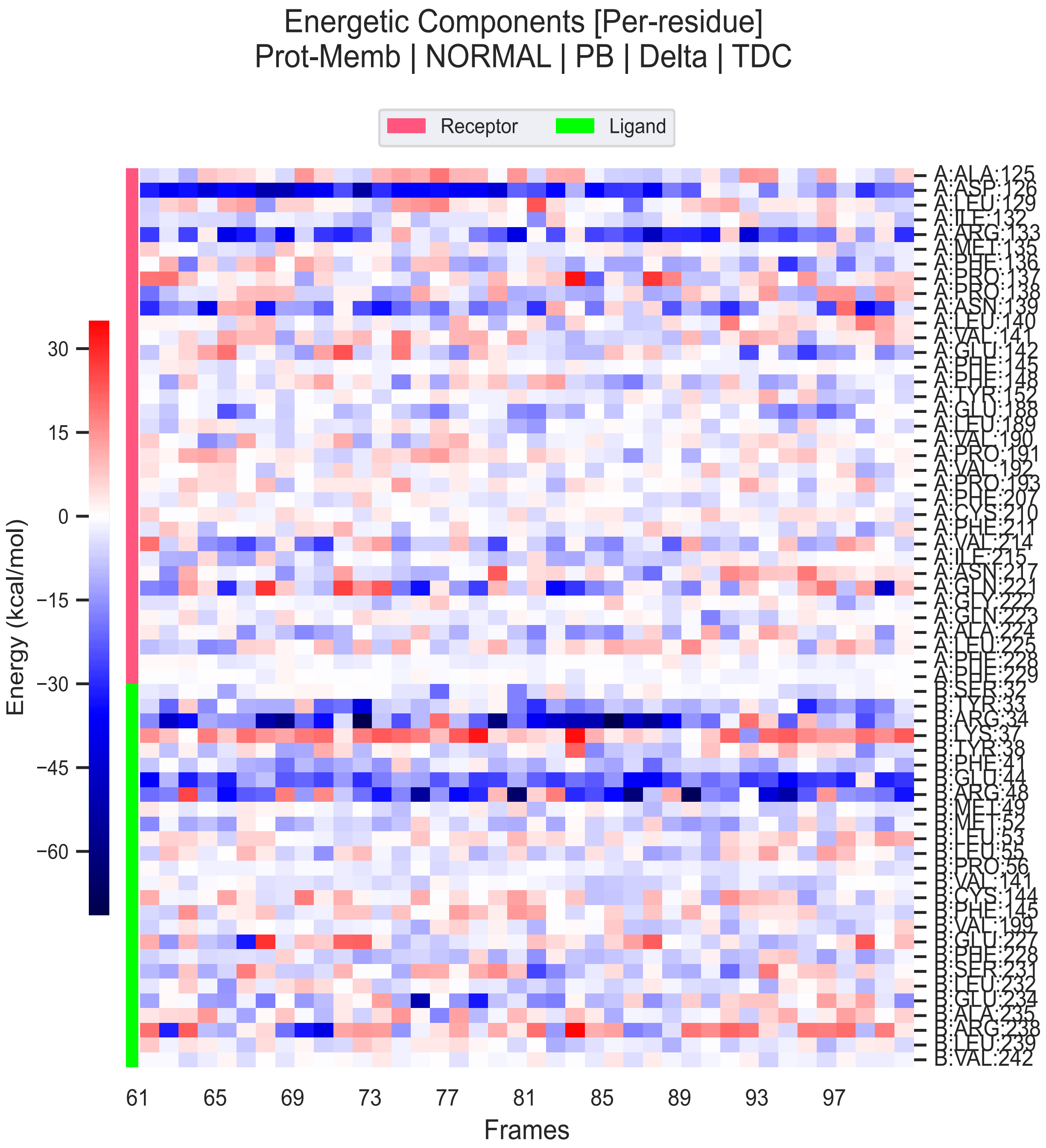

Figure S9. Residue contributions to the binding free energy of the canonical EAA1-EAA1 complex.

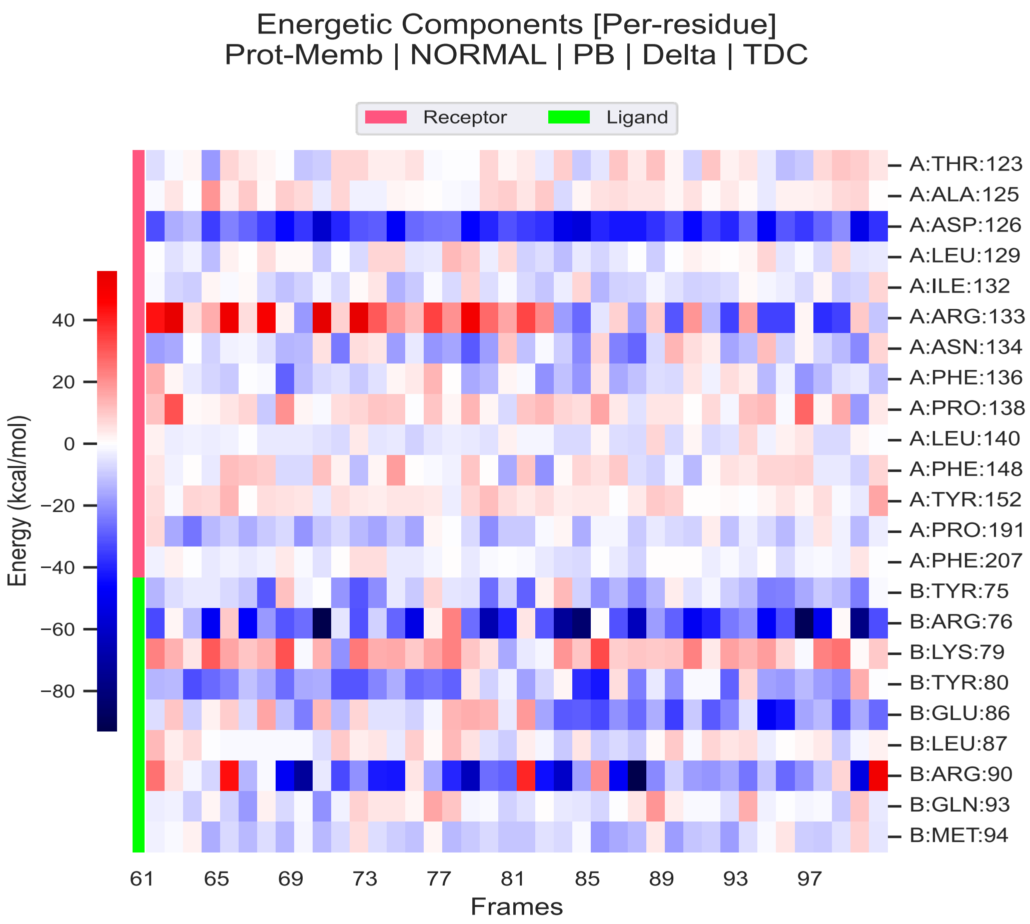

Figure S10. Residue contributions to the binding free energy of the E7EUV6-EAA1 complex.

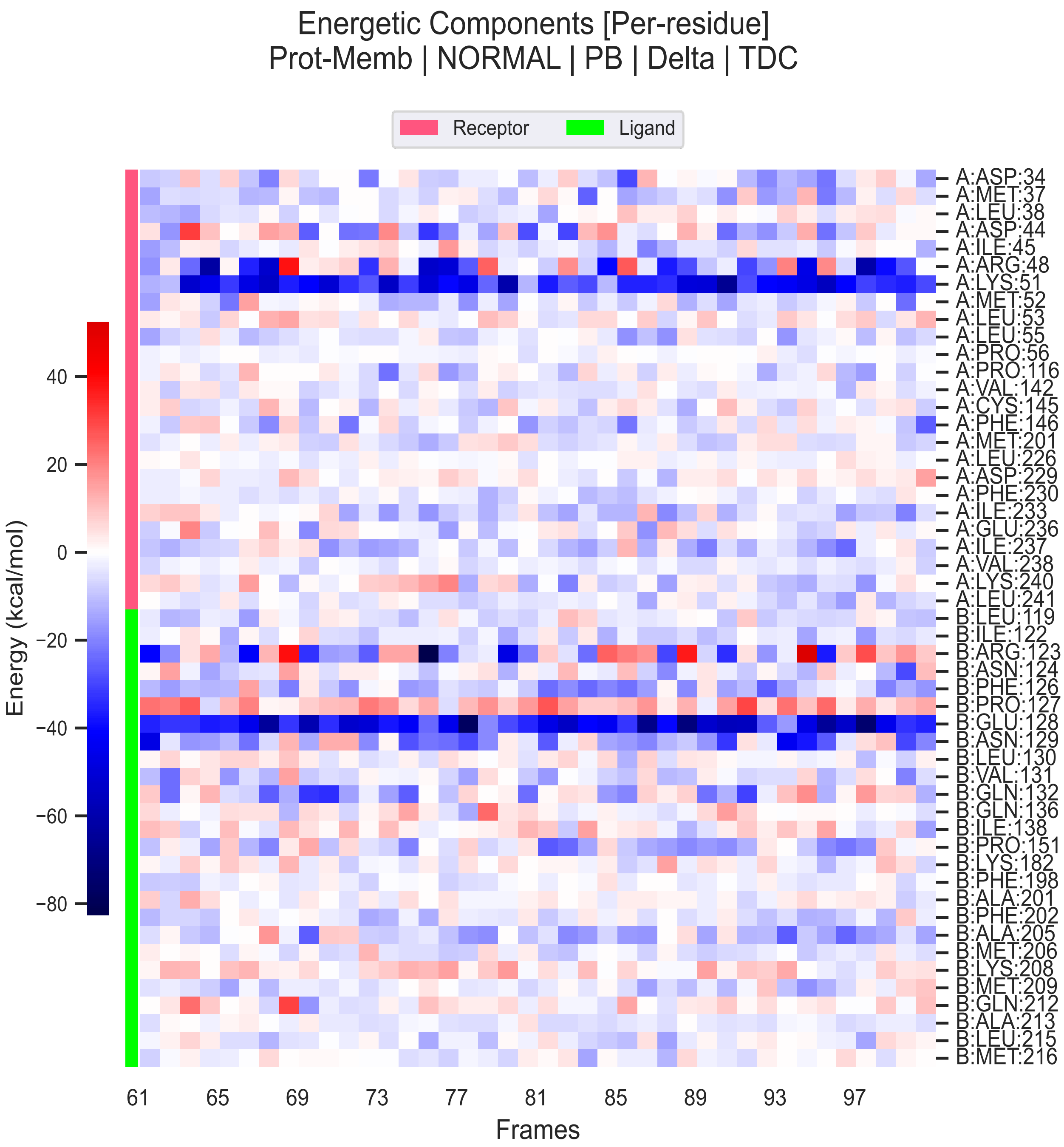

Figure S11. Residue contributions to the binding free energy of the A0A2R8Y642-EAA2 complex.

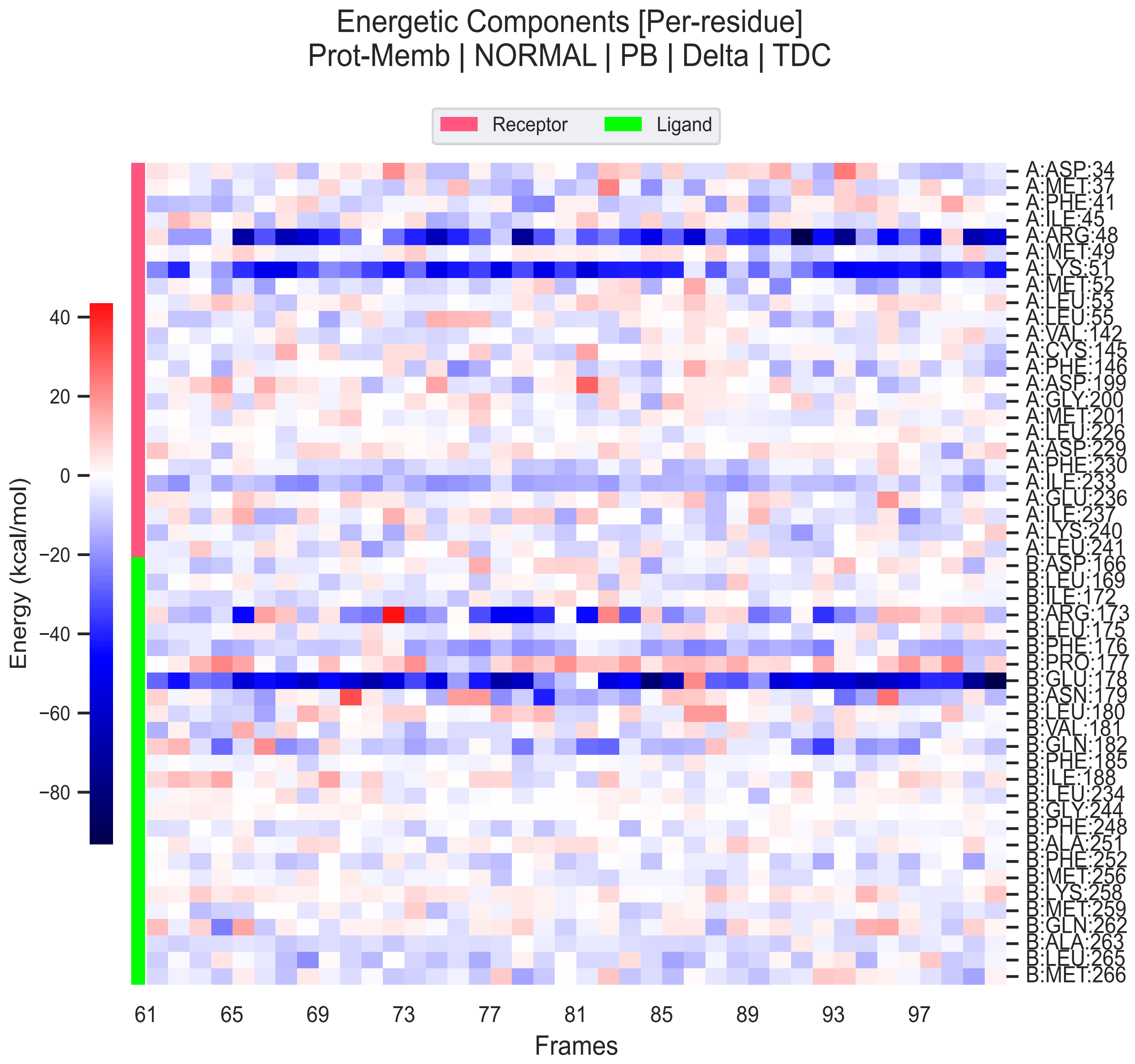

Figure S12. Residue contributions to the binding free energy of the canonical C9J9N5-EAA2 complex.

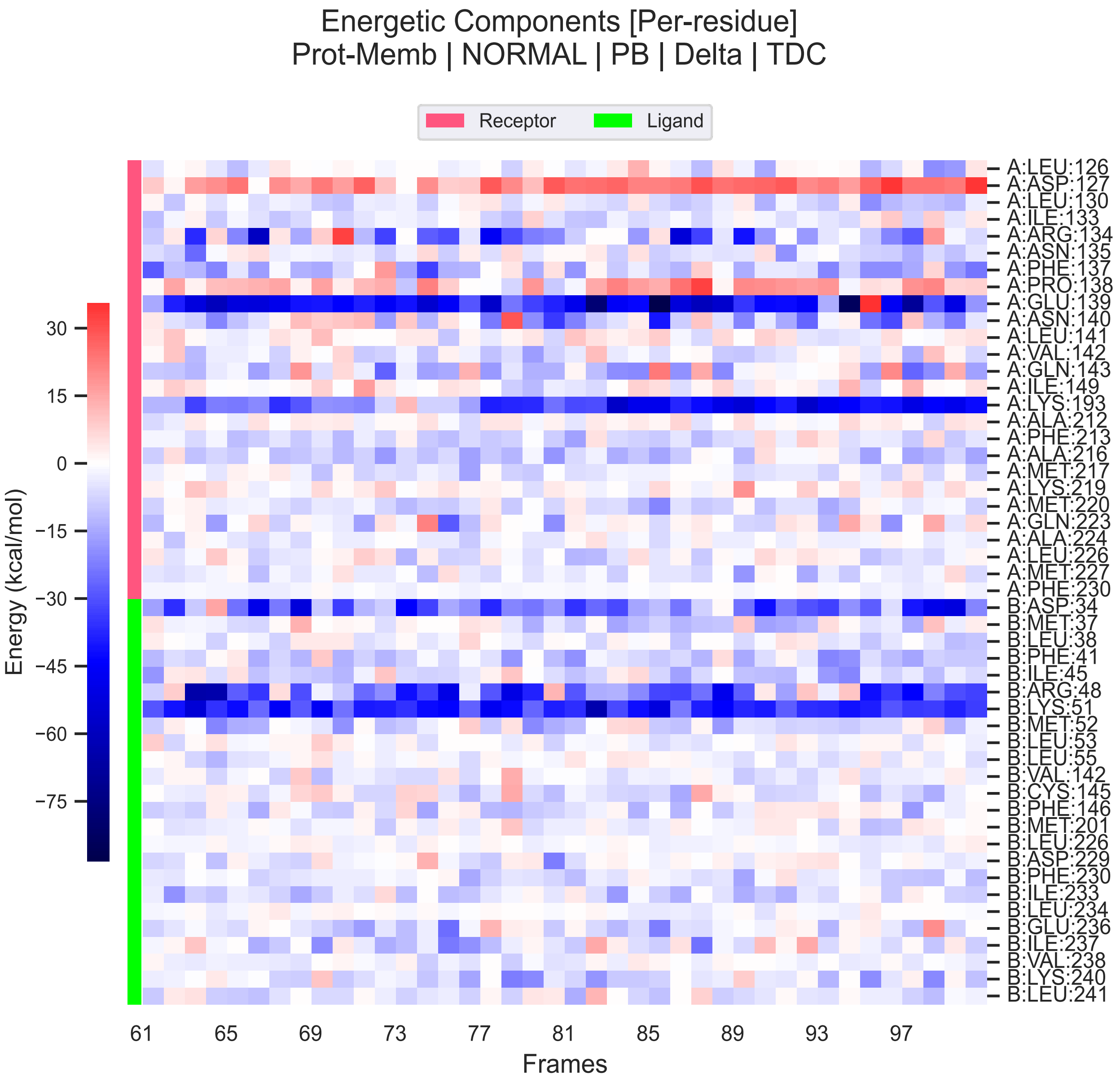

Figure S13. Residue contributions to the binding free energy of the canonical EAA2-EAA2 complex.

Table S9. Residue-wise decomposition of MMPBSA calculations of canonical EAA1 homodimer through 40 timestep MD (last 20ns)

EAA1 (receptor)		EAA1 (ligand)	
Residues	Count	Residues	Count
ARG:173	40	ARG:278	40
ASN:179	40	ARG:44	40
ASN:257	40	ARG:88	40
ASP:166	40	GLU:84	40
GLU:182	40	GLN:91	34
GLN:263	37	GLU:274	32
GLN:261	36	GLU:267	26
GLU:228	31	TYR:73	24
THR:163	20	LEU:93	17
GLY:262	14	LEU:272	16
VAL:181	12	VAL:181	15
LEU:229	11	LYS:77	14
LEU:169	5	LEU:279	7
ALA:165	4	LEU:85	6
ILE:255	4	SER:271	4
LEU:265	4	CYS:184	3
MET:258	4	VAL:239	3
PHE:247	4	ALA:275	2
ALA:264	3	MET:92	2
ILE:172	3	SER:72	2
LEU:180	3	ILE:276	1
VAL:254	2	PHE:185	1
PRO:178	1	PHE:268	1
Grand Total	398	Grand Total	370

Table S10. Residue-wise decomposition comparison of MMPBSA calculations of EAA1-E7EUV6 dimer through 40 timestep MD (last 20ns).

EAA1 homodimer (receptor)		EAA1-E7EUV6 dimer (receptor)	
Residues	Count	Residues	Count
ARG:173	40	ARG:173	40
ASN:179	40	ASN:174	40
ASN:257	40	ASN:179	40
ASP:166	40	ASP:166	40
GLU:182	40	THR:163	15
GLN:263	37	LEU:180	7
GLN:261	36	LEU:169	6
GLU:228	31	VAL:181	5
THR:163	20	ALA:165	3
GLY:262	14	ILE:172	3
VAL:181	12	VAL:246	1
LEU:229	11	-	-
LEU:169	5	-	-
ALA:165	4	-	-
ILE:255	4	-	-
LEU:265	4	-	-
MET:258	4	-	-
PHE:247	4	-	-
ALA:264	3	-	-
ILE:172	3	-	-
LEU:180	3	-	-
VAL:254	2	-	-
PRO:178	1	-	-
Grand Total	398	Grand Total	200

Table S11. Residue-wise decomposition of MMPBSA calculations of canonical EAA2 dimer through 40 timestep MD (last 20ns).

EAA2 (receptor)		EAA2 (ligand)	
Residues	Count	Residues	Count
ARG:173	40	ARG:87	40
ASN:174	40	ASP:268	40
ASN:179	40	ASP:73	40
ASP:166	40	GLU:275	38
GLU:178	39	LYS:90	24
GLN:182	38	LEU:273	22
LYS:232	36	LEU:280	15
GLN:262	35	LEU:77	10
VAL:142	18	LEU:92	10
ALA:263	12	LYS:279	9
LEU:265	11	VAL:277	7
ALA:255	9	LEU:255	3
ILE:172	7	MET:240	3
LEU:169	7	CYS:184	2
LEU:234	3	MET:91	2
MET:259	3	ILE:276	1
LEU:165	2	ILE:84	1
LEU:180	2	MET:76	1
MET:256	2	MET:88	1
ALA:251	1	PHE:185	1
LYS:258	1	-	-
MET:266	1	-	-
PHE:270	1	-	-
Grand Total	388	Grand Total	270

Table S12. Residue-wise decomposition comparison of MMPBSA calculations of EAA2_A0A2R8Y642 dimer through 40 timestep MD (last 20ns).

EAA2 homodimer (ligand)		EAA2_A0A2R8Y642 dimer (receptor)	
Residues	Count	Residues	Count
ARG:87	40	ARG:48	40
ASP:268	40	ASP:73	40
ASP:73	40	ASP:83	40
GLU:275	38	ASP:268	39
LYS:90	24	GLU:275	37
LEU:273	22	LYS:90	28
LEU:280	15	LEU:280	21
LEU:77	10	LEU:273	15
LEU:92	10	LEU:92	9
LYS:279	9	LYS:279	9
VAL:277	7	MET:240	8
LEU:255	3	VAL:181	7
MET:240	3	LEU:255	5
CYS:184	2	VAL:277	5
MET:91	2	LEU:77	3
ILE:276	1	MET:76	3
ILE:84	1	CYS:184	2
MET:76	1	PHE:185	2
MET:88	1	MET:88	1
PHE:185	1	MET:91	1
-	-	-	-
-	-	-	-
-	-	-	-
Grand Total	398	Grand Total	315

Table S13. Residue-wise decomposition comparison of MMPBSA calculations of EAA2_C9J9N5 dimer through 40 timestep MD (last 20ns).

EAA2 homodimer (ligand)		EAA2_A0A2R8Y642 dimer (receptor)	
Residues	Count	Residues	Count
ARG:87	40	ARG:87	40
ASP:268	40	ASP:238	40
ASP:73	40	ASP:268	40
GLU:275	38	ASP:34	40
LYS:90	24	GLU:275	37
LEU:273	22	LEU:92	21
LEU:280	15	LYS:90	21
LEU:77	10	LEU:273	19
LEU:92	10	LEU:280	14
LYS:279	9	LYS:279	10
VAL:277	7	VAL:181	5
LEU:255	3	LEU:255	4
MET:240	3	LEU:77	4
CYS:184	2	MET:240	4
MET:91	2	MET:76	4
ILE:276	1	VAL:277	4
ILE:84	1	CYS:184	2
MET:76	1	ILE:276	2
MET:88	1	MET:91	2
PHE:185	1	ILE:272	1
-	-	MET:88	1
-	-	-	-
-	-	-	-
Grand Total	398	Grand Total	315

Table S14. Residue-wise decomposition of MMPBSA calculations of A0A2R8Y4N0 homodimer through 40 timestep MD (last 20ns).

A0A2R8Y4N0 homodimer (receptor)		A0A2R8Y4N0 homodimer (ligand)	
Residues	Count	Residues	Count
ARG:285	40	ARG:285	40
ASN:133	40	ARG:327	40
ASN:288	40	ASN:288	40
ASP:23	40	ASP:23	40
GLU:30	39	LYS:329	39
ASP:280	38	GLN:291	38
GLN:108	37	GLU:30	36
GLN:17	36	GLN:17	34
GLN:291	36	ASP:280	31
GLU:132	36	GLU:328	31
GLY:193	20	LYS:13	26
THR:216	18	ALA:194	17
GLY:105	13	LEU:20	16
LEU:134	13	GLY:193	13
LEU:28	13	LEU:35	13
LEU:20	10	HSD:284	12
THR:210	9	THR:210	11
LEU:206	8	LEU:207	9
LYS:34	7	LEU:28	9
LEU:207	5	LYS:34	9
HSD:284	4	LEU:206	6
LEU:35	4	TYR:278	6
SER:287	4	VAL:203	3
MET:281	3	ALA:10	2
PHE:127	3	ILE:31	2
VAL:203	2	MET:281	2
ALA:10	1	PRO:124	2
ALA:194	1	ALA:6	1
MET:21	1	MET:14	1
PRO:124	1	PHE:25	1
SER:196	1	-	-
VAL:32	1	-	-
Grand Total	524	Grand Total	530

Table S15. Residue-wise decomposition of MMPBSA calculations of H0Y7R2 homodimer through 40 timestep MD (last 20ns).

H0Y7R2 homodimer (receptor)		H0Y7R2 homodimer (ligand)	
Residues	Count	Residues	Count
ARG:115	40	ARG:118	40
ARG:118	40	ASN:197	40
ASN:147	40	ASN:200	40
ASN:3	40	ASP:130	40
GLU:186	40	GLU:186	40
ASN:200	38	GLN:145	36
GLU:136	37	GLU:136	35
GLN:145	36	ASP:148	32
ASP:130	35	THR:132	27
ASP:150	35	THR:158	26
ASP:148	34	ASP:150	23
LEU:149	31	ALA:97	21
LEU:5	31	THR:9	21
LEU:151	30	LEU:5	20
THR:132	28	PHE:1	18
PHE:1	27	LEU:149	16
THR:9	23	THR:126	16
LEU:182	14	VAL:199	14
THR:126	14	MET:129	13
LEU:187	13	ALA:137	9
VAL:120	13	LEU:134	9
ILE:116	11	LEU:182	8
SER:96	9	VAL:120	8
ILE:198	8	GLY:154	7
VAL:199	8	SER:96	7
ALA:144	7	SER:6	6
ALA:97	7	ILE:127	5
VAL:13	7	ILE:116	4
SER:95	6	ALA:144	3
ALA:137	5	SER:183	3
ILE:127	4	SER:94	3
MET:190	3	VAL:13	3
SER:6	3	ALA:133	2

SER:94	3	ILE:16	2
ILE:153	2	MET:10	2
MET:129	2	MET:190	2
SER:183	2	ALA:140	1
A:LEU:5	1	ILE:153	1
ALA:133	1	VAL:123	1
ALA:140	1	VAL:141	1
ILE:24	1	-	-
LEU:134	1	-	-
VAL:123	1	-	-
VAL:141	1	-	-
Grand Total	733	Grand Total	605

a) A0A2R8Y4N0dimer

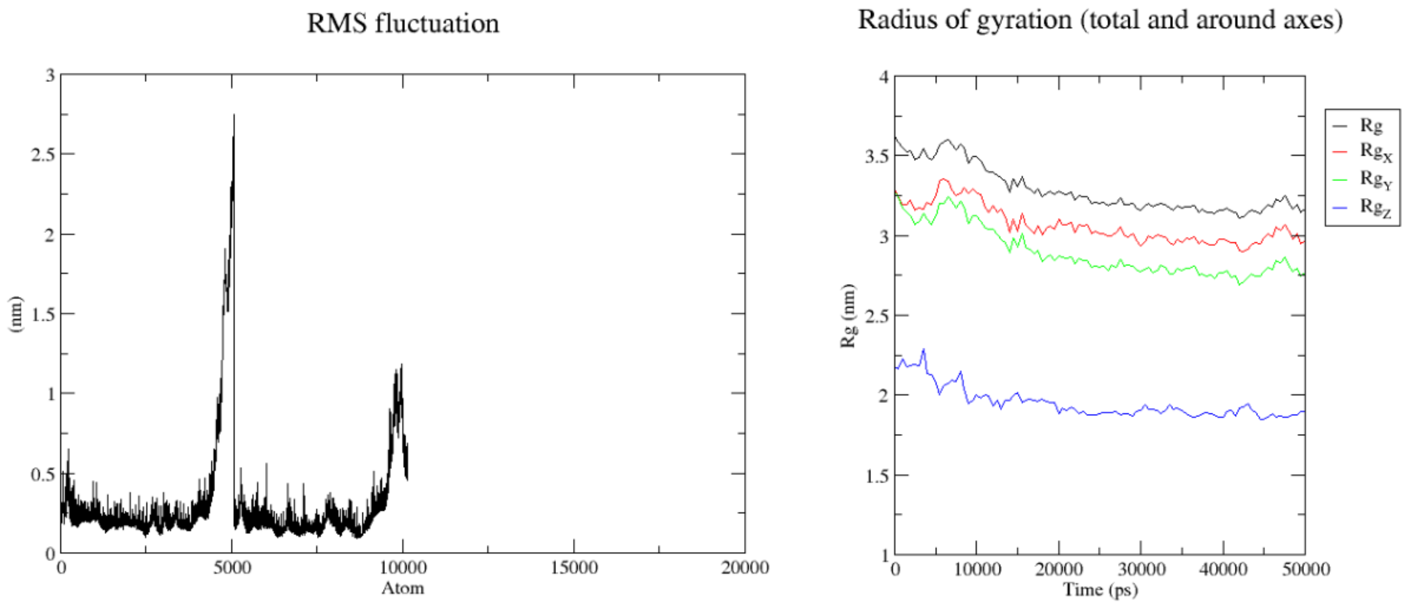

b) H0Y7R2dimer

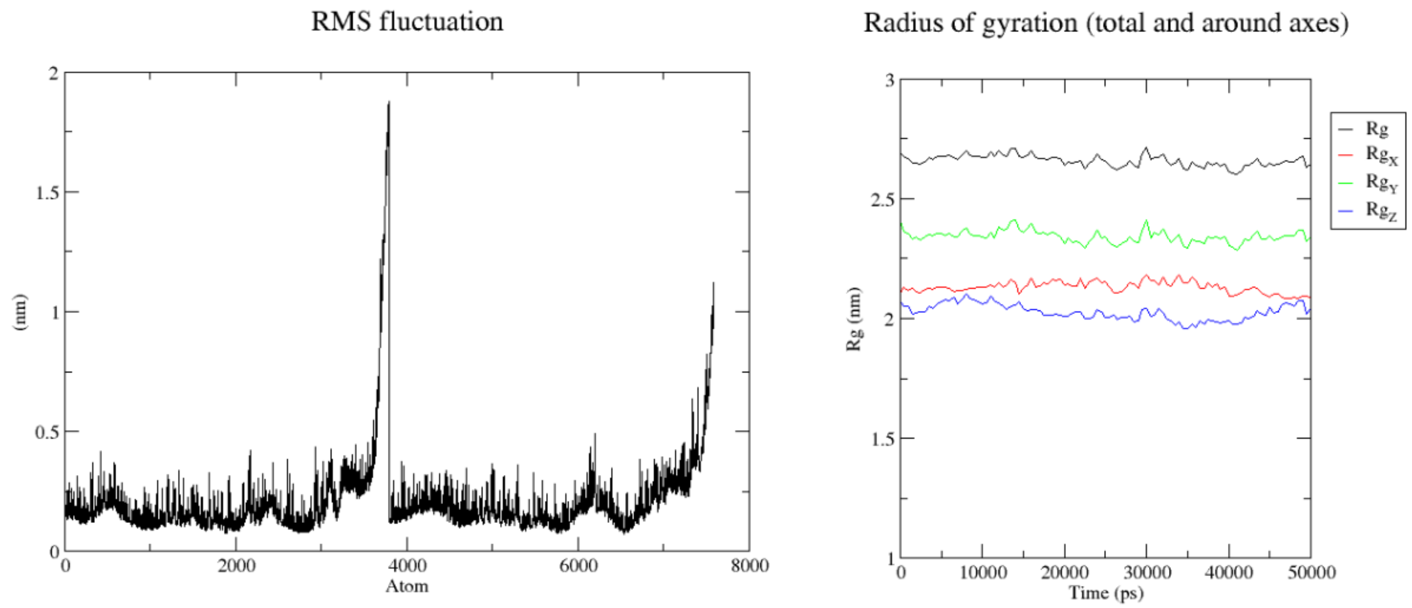

Figure S14. Residue-wise stability analysis of the sampled isoform dimers, A0A2R8YDN0 (a) and H0Y7R2 (b).
